# Supplementary material for: Structural basis for interaction between CLAMP and MSL2 proteins involved in the specific recruitment of the dosage compensation complex in Drosophila
Source: Nucleic Acids Res. 2022 Jun 1;50(11):6521–31. doi: 10.1093/nar/gkac455 (PMC9226498; doi:10.1093/nar/gkac455)
Supplement: gkac455_Supplemental_File [file gkac455_supplemental_file.pdf]

Supplemental information

Structural basis for interaction between CLAMP and MSL2 proteins involved in the specific recruitment of the dosage compensation complex in Drosophila

Evgeniya Tikhonova[1†], Sofia Mariasina[2,3†], Sergey Efimov[4], Vladimir Polshakov[2], Oksana Maksimenko[5], Pavel Georgiev[1*], Artem Bonchuk[1,5*]

[1]Department of the Control of Genetic Processes, Institute of Gene Biology, Moscow, 119334, Russia
[2]Center for Magnetic Tomography and Spectroscopy, Faculty of Fundamental Medicine, M.V. Lomonosov Moscow State University, Moscow 119991, Russia
[3]Faculty of Biology and Biotechnology, Higher School of Economics, Moscow 101000, Russia
[4]Kazan State University
[5]Center for Precision Genome Editing and Genetic Technologies for Biomedicine, Institute of Gene Biology, Moscow, 119334, Russia

† These authors contributed equally to the paper as first authors

* To whom correspondence should be addressed. Tel: +7(499)135-60-89 Fax: +7(499)135-41-05; Email: georgiev_p@mail.ru, bonchuk_a@genebiology.ru

SUPPLEMENTARY METHODS

Protein expression and purification.

BL21 (DE3) cells were transformed with vectors encoding cDNAs of CLAMP derivatives and $MSL2^{618\text{-}655}$ fused with TEV-cleavable Thioredoxin and 6xHis-tag. Cells were grown in 700 ml of LB medium at 37°C to OD=0.6-1.0, then fresh 100 mM stock of Zinc Acetate was added to final concentration of 0.2 mM and protein expression was induced with 1 mM IPTG overnight at 18°C.

Cells were disrupted by sonication in 15 ml of buffer A (30 mM HEPES-KOH pH 7.5, 400 mM NaCl, 5 mM β-mercaptoethanol, 10 mM Imidazole, 0.1 % NP40, 5% (v/v) Glycerol, 0.1 mM $ZnCl_2$) containing 1 mM PMSF and Calbiochem Complete Protease Inhibitor Cocktail VII (1μL/1ml). After centrifugation lysate was applied to Ni-NTA column, and after washing with 20 ml of (30 mM HEPES-KOH pH 7.5, 400 mM NaCl, 5 mM β-mercaptoethanol, 30 mM Imidazole) protein was eluted with 15 ml of (30 mM HEPES-KOH pH 7.5, 400 mM NaCl, 5 mM β-mercaptoethanol, 300 mM Imidazole).

For cleavage of Thioredoxin-6xHis-tag sodium citrate was added to final concentration of 5mM, $ZnCl_2$ to 0.01 mM and 6x-His-tagged TEV protease was added at molar ratio approximately 1:50 directly to the eluted protein, mixture was incubated for 2 hours at room temperature and dialyzed overnight at 4°C against degassed 30 mM HEPES-KOH pH 7.5, 400 mM NaCl, 1 mM β-mercaptoethanol, 10 mM Imidazole, 0.1 mM $ZnCl_2$, then filtered and applied to Ni-NTA column, flowthrough was collected, dialyzed against degassed 20 mM Tris-HCl, pH 7.4, 0.1 mM $ZnCl_2$, 1 mM DTT and further purified using SOURCE15Q 4.6/100 column (GE Healthcare). Proteins were either eluted with 0-500 mM NaCl gradient (CLAMP deletion derivatives), or collected as flowthrough ($MSL2^{618\text{-}655}$). Sample homogeneity was confirmed with size-exclusion chromatography which was performed using Superdex 200 10/300GL column (GE Healthcare) in 20 mM Tris-HCl, pH 7.4, 150 mM NaCl, 0.1 mM $ZnCl_2$, 1 mM DTT.

Fly crosses and transgenic lines

Drosophila strains were grown at 25°C under standard culture conditions. The transgenic constructs (Ubi-clamp or Ubi-MSL2) were injected into preblastoderm embryos using the φC31-mediated site-specific integration system at locus 86Fb (1). The emerging adults were crossed with the *y ac* w^{1118} flies, and the progeny carrying the transgene in the 86Fb region were identified by y^+ pigmented cuticle.

To assess the viability of transgenic lines expressing different CLAMP variants (CLAMP*), virgin $clamp^2$/CyO, GFP; Ubi:clamp*-HA/Ubi:clamp*-HA females were crossed with $clamp^2$/CyO, GFP; Ubi:clamp*-HA/Ubi:clamp*-HA males. Viability of transgenic flies expressing CLAMP* was calculated as the ratio of the homozygous males or females ($clamp^2/clamp^2$; Ubi:clamp*-HA/Ubi:clamp*-HA) relative to heterozygous male or females ($clamp^2$/CyO; Ubi:clamp*-HA/Ubi:clamp*-HA) divided by two.

To assess the *in vivo* role of mutations in MSL2, the viability of females homozygous for the Ubi:msl2*-FLAG transgene was assessed in homozygous transgenic lines (Ubi:msl2*-FLAG/Ubi:msl2*-FLAG), where msl2* expresses WT or one of the mutant variants of MSL2. The percentage of female viability with different Ubi:msl2*-FLAG transgene was estimated by taking the viability of males as 100%.

Fly protein extracts were performed as described (2).

Polytene chromosome staining

Drosophila 3rd instar larvae were cultured at 18°C under standard conditions. Immunostaining of polytene chromosomes was performed as described (3). The following primary antibodies were used: rabbit anti-MSl1 at 1:500 dilution, rabbit anti-Msl2 at 1:500 dilution, and monoclonal mouse anti-FLAG at 1:50 dilution. The secondary antibodies were Alexa Fluor 488 goat anti-mouse 1:2000 and Alexa Fluor 555 goat anti-rabbit 1:2000 (Invitrogen). The polytene chromosomes were co-stained with DAPI. Images were acquired on the Nikon Eclipse T*i* fluorescent microscope using Nikon DS-Qi2 digital camera, processed with ImageJ 1.50c4 and Fiji bundle 2.0.0-rc-46. 3-4 independent stainings and 4-5 samples of polytene chromosomes were performed with each transgenic line.

RNA isolation and quantitative analysis

Total RNA was isolated from 2- to 3-day-old adult females and males (used as a positive control) using the TRI reagent (Molecular Research Center, United States) according to the manufacturer's instructions. RNA

was treated with two units of DNase I (ThermoFisher) for 30 min at 37°C to eliminate genomic DNA and additionally purified with TRI reagent. The synthesis of cDNA was performed using 1 μg of RNA, 200 U of Protoscript II reverse transcriptase (NEB), RNAse inhibitor (Thermo Fisher), 0.5 mM dNTPs and 3 μM of random hexamers as a primer. The amounts of specific cDNA fragments were quantified by real-time PCR with EvaGreen (Biotium). At least four independent measurements were made for each RNA sample. Relative levels of mRNA expression were calculated in the linear amplification range by calibration to a standard genomic DNA curve to account for differences in primer efficiencies. Individual expression values were normalized with reference to RpL32 mRNA.

Analysis of C2H2 zinc-fingers amino-acid composition

We compared amino acid residues of the CLAMP N-terminal zinc finger at DNA-binding positions with their average abundance in C2H2 zinc-fingers. We developed a hidden Markov model of the C2H2 domain sequence based on 109192 representative domains from the Pfam database (4). We used it to calculate each residue's probabilities at given positions (see Supplementary Figure S7). The probability of each residue in a completely random sequence would be 5%. The CLAMP N-terminal zinc finger has histidine at -1 position, leucines at +1 and +3, alanines at +2 and +6 (+2 and +6 positions are not conserved in some species, substituted for threonine or histidine, respectively). Histidine at -1 position is found here in 4.8% of zinc fingers according to our model; leucine at +1 is rarely used for DNA recognition (5) but is present at this position in 7.2% of zinc fingers. Alanine or threonine at +2 is found in 11.2% zinc-fingers. Leucine is rarely found at position +3 (3.3% of all zinc fingers), a polar residue in most (93.3%) zinc fingers. Residue at +6 position also most often is polar (78.7%), but alanines (and histidines, which are found here in some species) sometimes (6.1% altogether) are present at this position (6,7). The first residue of the second beta-sheet often makes non-specific contacts with backbone phosphate, and arginine or lysine is usually (75.6%) found at this position (8), the CLAMP N-terminal zinc-finger has nontypical hydrophobic leucine at this position, which is found here only in 0.9% of zinc fingers. De novo prediction of the target site (9) of the CLAMP N-terminal zinc finger yields low-score, ambiguous results (the position-weight matrix is shown in Supplementary Figure S8). Altogether, these analyses suggest that the CLAMP N-terminal zinc-finger is very uncommon as a DNA-binding zinc finger and is most likely not involved in DNA-binding.

NMR Spectroscopy

The standard triple resonance experiments were performed for the sequential assignments of the backbone ^{1}H, ^{13}C and ^{15}N nuclei of both, CLAMP$^{87\text{-}153}$ and MSL2$^{618\text{-}655}$ protein fragments: HNCO, HN(CA)CO, CBCA(CO)NH, and HNCACB. Additionally, for the side-chain assignment and structure calculation, the ^{13}C-HCCH-TOCSY, DQF-COSY, ^{13}C-^{1}H HSQC-NOESY, and ^{15}N-^{1}H HSQC-NOESY spectra were collected for CLAMP$^{87\text{-}153}$. To study protein interaction and protein folding we measured ^{15}N-^{1}H HSQC or ^{15}N-^{1}H SOFAST HMQC spectra.

The acquired data were processed using NMRPipe (10), and analyzed using NMRFAM-Sparky software (11).

H/D exchange experiment

The H/D exchange experiment was performed using freeze-dried ^{15}N labelled Clamp$^{87\text{-}153}$ sample (0.3 mM) by dissolving it in 100% D_2O. The ^{15}N,^{1}H SOFAST HMQC spectrum was measured as soon as possible during 24 minutes. The analysis of resulting spectrum was performed by comparison with the same spectrum measured for sample dissolved in 5%D_2O/95% H_2O.

NMR chemical shifts assignments and data deposition

Backbone ^{1}H, ^{13}C and ^{15}N resonance assignment was performed manually using NMRFAM-Sparky (11). Backbone amide ^{1}H and ^{15}N resonance assignments of CLAMP$^{87\text{-}153}$ and MSL2$^{618\text{-}655}$ were achieved for all non-proline residues. Side-chain signals of CLAMP$^{87\text{-}153}$ were assigned manually using the information on the backbone assignments. The ^{1}H, ^{15}N and ^{13}C chemical shifts of CLAMP$^{87\text{-}153}$ and the backbone assignments of MSL2$^{618\text{-}655}$ have been deposited into the BioMagResBank (www.bmrb.wisc.edu) under the accession numbers BMRB-34600 and BMRB-51286, correspondingly.

Protein chain mobility, secondary structure elements and relaxation

The protein chain mobility and secondary structure elements of CLAMP and MSL2 were identified with TALOS+ program using ^{13}Cα, ^{13}Cβ, ^{13}C′ and ^{15}N chemical shifts (12). These data were also used to get backbone torsion angle restraints for structure calculations.

Correlation time of protein tumbling was calculated from the NMR relaxation data. The obtained values were used to estimate protein radius using the Stokes' law:

$$\tau_c = \frac{4\pi\eta r^3}{3kT}$$

Estimation was done with the value 8.90×10^{-4} Pa for the water viscosity at 25 °C (13)

NMR Restraints generation and Structure calculation

The solution structure of $CLAMP^{87\text{-}153}$ was calculated using following set of restraints: (1) distance restraints obtained from ^{13}C-^{1}H HSQC-NOESY and ^{15}N-^{1}H HSQC-NOESY spectra; (2) torsion angle restraints obtained from chemical shifts using TALOS+ program; (3) H/D exchange rates (Supplementary Table S1A).

The structure calculation was performed using ARIA (14) and CNS (15) programs. Structural statistics is shown at Supplementary Table S1A and B. Ramachandran plot analysis indicates that all non-glycine residues are falling into favorable regions.

CLAMP N-terminal C2H2 domain is a classic example of zinc-finger in terms of both fold and metal-binding environment (Figure 1A). Its 26-residue sequence adopts a ββα tertiary fold consisting of short β-hairpin (β_1:F127-C129/β_2:S133-F136) followed by long C-terminal α-helix (α_3:L139-T150) which is in agreement with the secondary structure calculation based on chemical shifts. The fold is further stabilized by a hydrophobic core that is formed around the side-chain of L142 residue. The metal coordination site consists of two cysteine and two histidine residues (C_2H_2).

H/D exchange experiment revealed three H^N atoms having much slower exchange rate comparing to the others (Supplementary Figure S2). One of them, F136 H^N, is placed in β-hairpin therefore its slow exchange rate was interpreted as a result of strong H-bond with carbonyl group of F127 and used to get two H-bond restraints. The other two H^N atoms, namely V131 and C132, are not placed in β-sheet region and therefore their slow exchange rates could not be explained by participating in H-bonds. These residues are near zinc ion, and can be affected by its shielding effect. Upstream of Zinc-finger, CLAMP is unstructured, and the presence of upstream fragment has no impact on Zinc-finger structure. It was demonstrated by expression of ^{15}N-labelled CLAMP fragments: $CLAMP^{1\text{-}153}$ and $CLAMP^{40\text{-}153}$, with the subsequent HSQC spectra measurement (Supplementary Figure S3).

Molecular modeling of $MSL2^{618\text{-}655}$ structure was performed based on chemical shifts using CS-Rossetta (16). The modelling suggests a possibility of β-hairpin formation at V634−N638 and G641−N647. At the same time, the order parameter S2 for these residues does not exceed 0.7 (Supplementary Figure S12), which corresponds to unstructured protein chain. To validate the formation of β-hairpins in $MSL2^{618\text{-}655}$ we assigned Hα-atoms using HNHA and HBHA(CO)NH spectra and measured 3D 15N-1H HSQC-NOESY spectra, but no NOE between V634−N638 and G641−N647 were found. Thus, we approved the absence of β-hairpins in the corresponding region.

Chemical Shifts perturbation

Interaction of CLAMP with MSL2 was studied using NMR titration experiments. ^{15}N-labelled $MSL2^{618\text{-}655}$ at a concentration of 0.1-0.5 mM were used. The unlabelled $CLAMP^{87\text{-}153}$ concentration increased from 1:1 to 1:13 protein–protein ratio. For each titration point, a ^{15}N–^{1}H SOFAST HMQC spectrum (17) was recorded.

The titration of ^{15}N-labeled CLAMP by MSL2 were performed using $CLAMP^{87\text{-}153}$, $CLAMP^{40\text{-}153}$, and $CLAMP^{1\text{-}153}$ constructs (0.01-0.50 mM) and unlabelled $MSL2^{618\text{-}655}$ (0.00-0.54 mM).

The chemical shift perturbation data were calculated using the formula: $((\Delta\delta(^{1}H^{i}))^2 + (\Delta\delta(^{15}N^{i})/25)^2)^{½}$.

The value of K_d were estimated from NMR titration experiments carried out at 25 °C (Supplementary Figure S14). 26 ^{1}H amide resonances were used in non-linear fitting of K_d values by the following equation (18):

$$\Delta\delta_{obs} = \frac{\Delta\delta_{max}}{2[P]_0}\left[(K_d + [P]_0 + [L]_0) - \sqrt{(K_d + [P]_0 + [L]_0)^2 - 4[P]_0[L]_0}\right]$$

where P_0 and L_0 are the total concentrations of CLAMP and the MSL2 in each titration step, $\Delta\delta_{obs}$ is the change of chemical shift value, and $\Delta\delta_{max}$ is the maximum change of the chemical shift accepted by the difference between the signal in free protein and protein in the presence its partner in the maximum concentration.

SUPPLEMENTARY TABLES

Supplementary Table S1. Statistics for the ensemble of the calculated 20 structures of the $CLAMP^{87-153}$. No NOE or dihedral angle violations are above 0.5 Å and 5° respectively.

A. Restraints used in the structure calculation

Total NOEs	355	Total dihedral angles	40
Long range (|i − j|> 4)	30	Phi (ϕ)	20
Medium (1 < |i − j|≤ 4)	43	Psi (ψ)	20
Sequential (|i − j|= 1)	91	H-bonds	1
Intraresidue	191		

B. Restraint violations and structural statistics (for 20 structures)

Average RMSD	ensemble of 20 final structures	representative structure
From experimental restraints		
Distance (Å)	0.023±0.002	0.026
Dihedral (°)	0.57±0,12	0.49
From idealized covalent geometry		
Bonds (Å)	0.0018±0,0001	0.002
Angles (°)	0.397±0,014	0.414
Impropers (°)	0.295±0,014	0.272
Ramachandran plot statistics		
% of residues in most favorable region of Ramachandran plot	79.5	81.0
% of residues in disallowed region of Ramachandran plot	0	0

C. Superimposition on the representative structure (Å)

Backbone (C, Cα, N) RMSD over the structured protein core (residues 126-150)	0.46±0.10

Supplementary Table S2. (A) Comparison of the viability of males and females upon rescue of the clamp^2/clamp^2 and clamp^2/+ mutant background with CLAMP proteins expressed in transgenic constructs. **(B)** Comparison of the viability of males and females upon ectopic expression of MSL2 proteins.

A

	clamp^2/+		clamp^2/clamp^2	
	♂	♀	♂	♀
CLAMP^{WT}	246	224	67	64
	143	139	37	38
CLAMP^{K146E}	68	73	2	1
	87	83	2	2
	112	109	3	4
CLAMP$^{L139A\ K146E}$	131	134	4	5
	72	81	2	1
	67	69	2	1
CLAMP$^{K146E\ R147E}$	66	65	4	5
	39	35	3	2
	111	117	6	6

B

	♀	♂
MSL2^{WT}	1	8
	0	12
	2	23
MSL2^{Y643A}	56	64
	49	56
	84	93
MSL2$^{E639A;Y643A}$	80	78
	91	88
	82	91
MSL2$^{L633A;V634A;E639A;Y643A}$	107	106
	87	87
	82	77
MSL2^{dvCBD}	31	82
	35	91
	29	77
MSL2$^{dvCBD+\Delta CXC}$	140	132
	95	89
	74	71

Supplementary Table S3. Oligonucleotides used for cloning and real-time PCR. Restriction enzyme sites are shown in small letters, the corresponding enzymes are noted. Nucleotide substitutions in mutagenic primers are also shown in small letters.

Primers for cloning	Direct	Reverse	REs
CLAMP 87-153	CTGgaattcATGGAAGACCTTACCAA	AACgtcgacTTCCCCGTCTGTATGCAT	*Hinc*II,*Sal*I
CLAMP 40-153	CTGgaattcATGAAAACGGAGCAGCAGC	AACgtcgacTTCCCCGTCTGTATGCAT	*Eco*RI,*Sal*I
MSL2 618-655	TCTggatccATAAGCCTAGTGCCGC	GTGgaattcCTAATCAAGGGGCT	*Bam*HI, *Eco*RI
MSL2	CGAGTACTGGTGGCGTGGGTCGGACCGAC	TCTgtcgacCAAGTCATCCGAGCCCGACA	*Sca*I, *Sal*I
CLAMP 1-153	CTGgaattcATGGAAGACCTTACCAA	AACgtcgacTTCCCCGTCTGTATGCAT	*Eco*RI,*Sal*I
CLAMP 1-153 ^{I122A}	CTAACACCgcATCCAACATAAGC	CTTATGTTGGATgcGGTGTTAGC	
CLAMP 1-153 ^{H138A}	GTTCCCTgcTTTGGCACTTC	GAAGTGCCAAAgcAGGGAACAT	
CLAMP 1-153 ^{L139A}	CCCTCATgcGGCACTTCTTAATG	CATTAAGAAGTGCCgcATGAGGGAAC	
CLAMP 1-153 ^{L141A}	CATTTGGCAgcTCTTAATGCTC	GAGCATTAAGAgcTGCCAAATG	
CLAMP 1-153 ^{N143A}	CACTTCTTgcTGCTCATGAGGAG	CTCATGAGCAgcAAGAAGTGCC	
CLAMP 1-153 ^{K146E}	CTCATgAGCGGATGCATACAGAC	GTATGCATCCGCTcATGAGCATTAAG	
CLAMP 1-153 ^{R147E}	GCTCATAAGgaGATGCATACAGACG	GTCTGTATGCATCtcCTTATGAGC	
CLAMP 1-153 ^{K146ER147E}	GTCCATgAGgaGATGCATACAGACG	GTCTGTATGCATCtcCTCATGAGC	
CLAMP 1-153 ^{N143AK146ER147E}	GCTCATgAGgaGATGCATACAGACG	CTCATGAGCAgcAAGAAGTGCC	
HA-CLAMP	CTGgaattcATGGAAGACCTTACCAA	TAGgtcgacCTATAACCCACCGATAATC	*Eco*RI,*Sal*I
MSL2-FLAG	TTCgatatcATGGCCCAGACGGCATACTT	TCTcccgggCAAGTCATCCGAGCCCGA	*Eco*RV,*Sma*I
MSL2-FLAG ^{Y643A}	AAGGCGAGgcCCAGGGCTTCAATATCTT	AGCCCTGGgcCTCGCCTTTCTCATTCTG	
MSL2-FLAG ^{E639AY643A}	TCAGAATGcGAAAGGCGAGgcCCAGG	CGCCTTTCgCATTCTGAACAAGCACC	
MSL2-FLAG ^{L633AV634A} ^{E639AY643A}	CATCCTgcGGcGCTTGTTCAGAAT	AACAAGCgCCgcAGGATGCTGGGA	
amMSL2	CAAggatccATGAATGCCACAAGTCTTTACG	CTTgtcgacTCACACATCAATTTGTATATCACTGTC	*Bam*HI, *Sal*I
amCLAMP $^{1-204}$	GACggatccATGGTCAAAGGCAACACATC	TTGgtcgacTTATTGAAGATTACTAGGTGTTGTATTATG	*Bam*HI, *Sal*I
amMSL2 $^{338-432}$	GGTggatccATTCGACCACATATACCTGAATTAC	CTTgtcgacTCACACATCAATTTGTATATCACTGTC	*Bam*HI, *Sal*I

Primers for RT-qPCR	Direct	Reverse	
RoX1	CTTGTGCTTTCTCCTGAATGTG	TGTATTAGGCGGAGCTTCTTG	
RoX2	TTCGAAACGTTCGCCGAAGC	AGTCGTACTCATCTCACTGTCC	
RpL32	GTTCGATCCGTAACCGATGT	CCAGTCGGATCGATATGCTAA	

SUPPLEMENTARY FIGURES

Supplementary Figure S1. Protein chain mobility of CLAMP$^{87-153}$ represented by RCI-derived order parameter S^2 obtained from chemical shifts and the ANN-predicted secondary structure. Data obtained using Talos+ program (12).

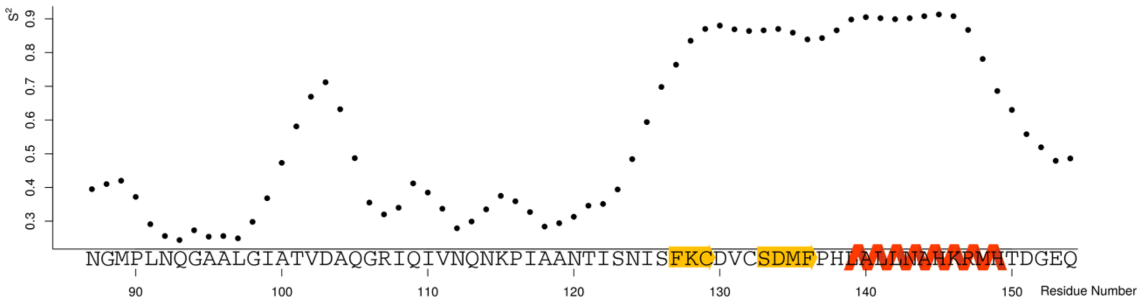

Supplementary Figure S2. H/D exchange experiment. ^{15}N,^{1}H SOFAST HMQC spectrum of ^{15}N CLAMP$^{87\text{-}153}$ dissolved in 5%D_2O/95% H_2O (blue) overlapped by spectrum of ^{15}N CLAMP$^{87\text{-}153}$ dissolved in 100%D_2O measured during first 20 minutes after dissolving (green). The signals of residues V131, C132 and F136 demonstrating longer H/D exchange rate are labeled. The spectrum was recorded using NMR spectrometer Bruker Avance 600 MHz at 298K.

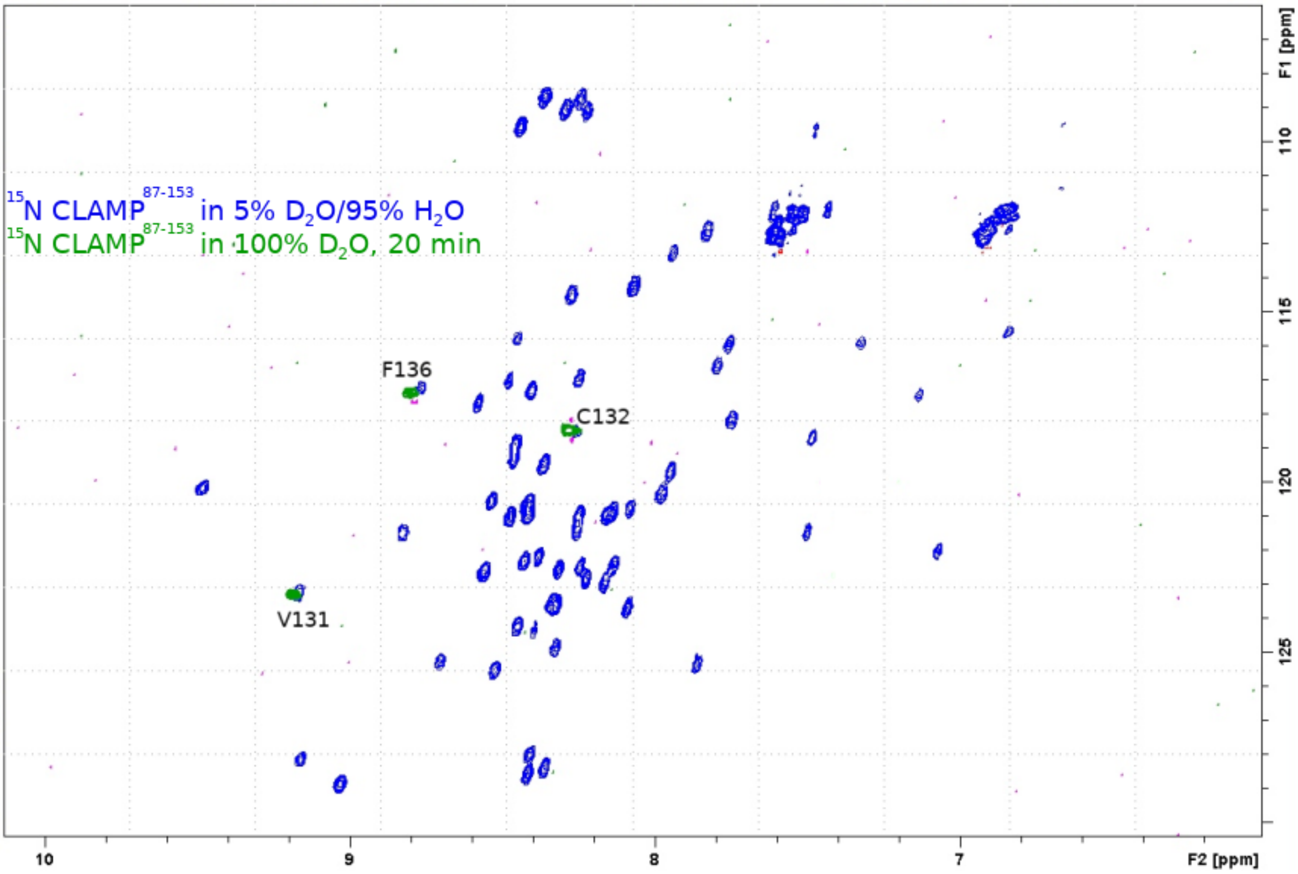

Supplementary Figure S3. ^{15}N,^{1}H SOFAST HMQC spectra of different fragments of ^{15}N-labelled CLAMP. Partial amino acid assignment (performed for residues 87-153) is shown. **(A)** Overlay of CLAMP$^{40\text{-}153}$ over CLAMP$^{87\text{-}153}$. **(B)** Overlay of CLAMP$^{1\text{-}153}$ over CLAMP$^{40\text{-}153}$. The spectra were recorded using NMR spectrometer Bruker Avance 600 MHz at 298K.

(A)

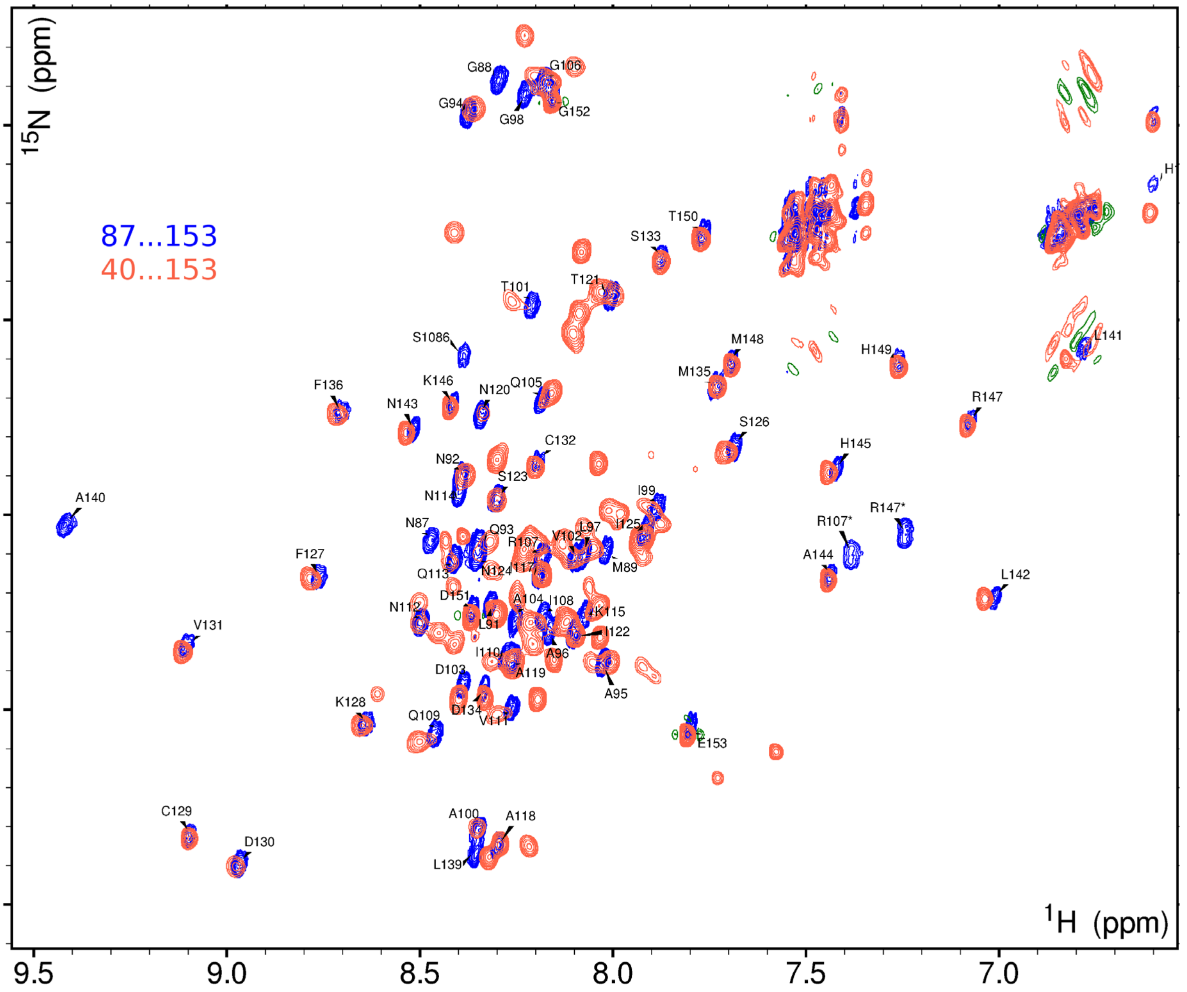

(B)

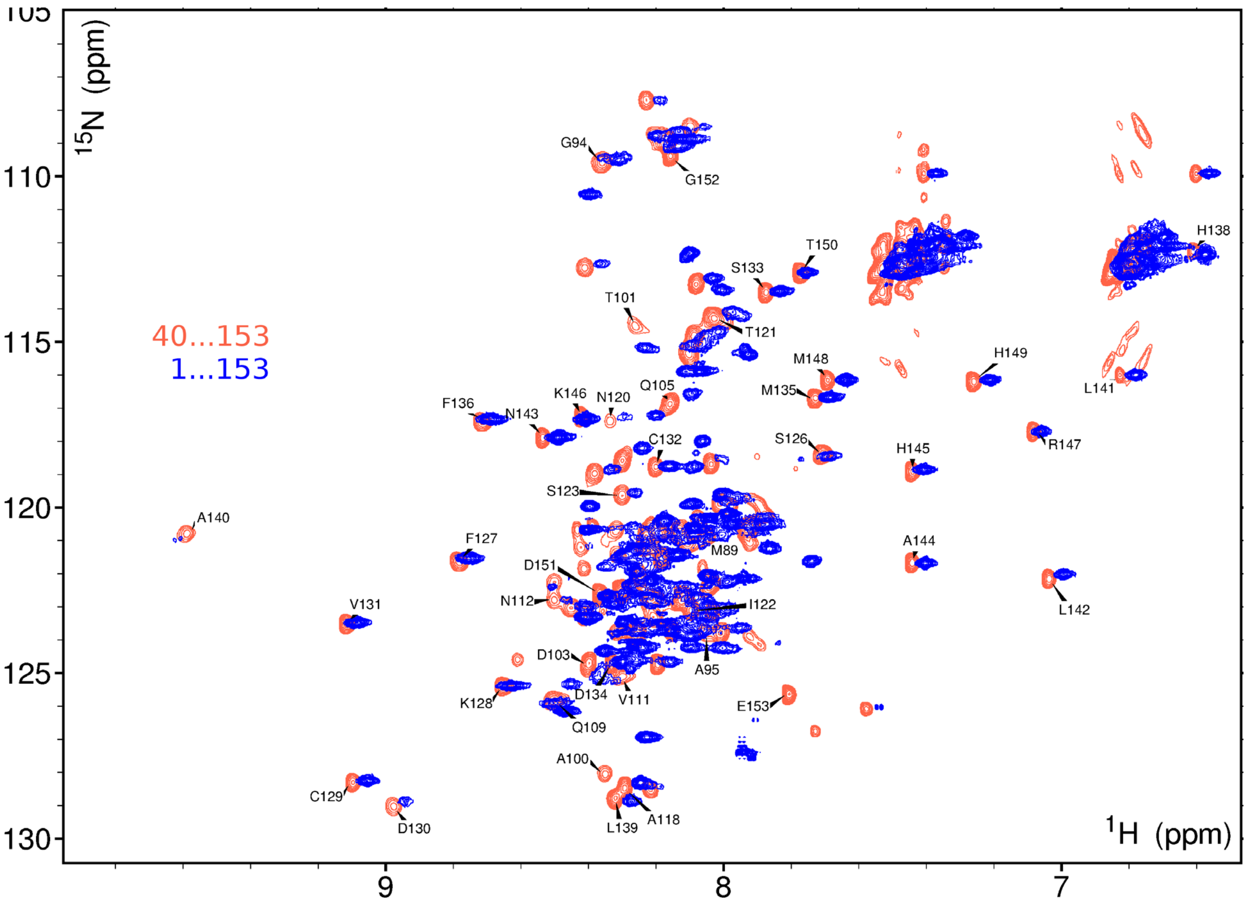

15N (ppm)
105
110
115
120
125
130
40...153
1...153
G94
G152
T150
S133
H138
T101
T121
M148
H149
L141
K146
N120
Q105
M135
F136
N143
C132
S126
H145
R147
S123
A140
F127
M89
A144
D151
N112
I122
L142
V131
D103
A95
D134
V111
K128
E153
Q109
A100
C129
D130
L139
A118
1H (ppm)
9
8
7

Supplementary Figure S4. Multiple sequence alignment of MSL2-interacting CLAMP N-terminal region from various insects. Position of zinc-finger domain is shown. Identical residues are shown in yellow, blocks of conserved – in blue. Phylogenetic positions of taxa are shown according to (19).

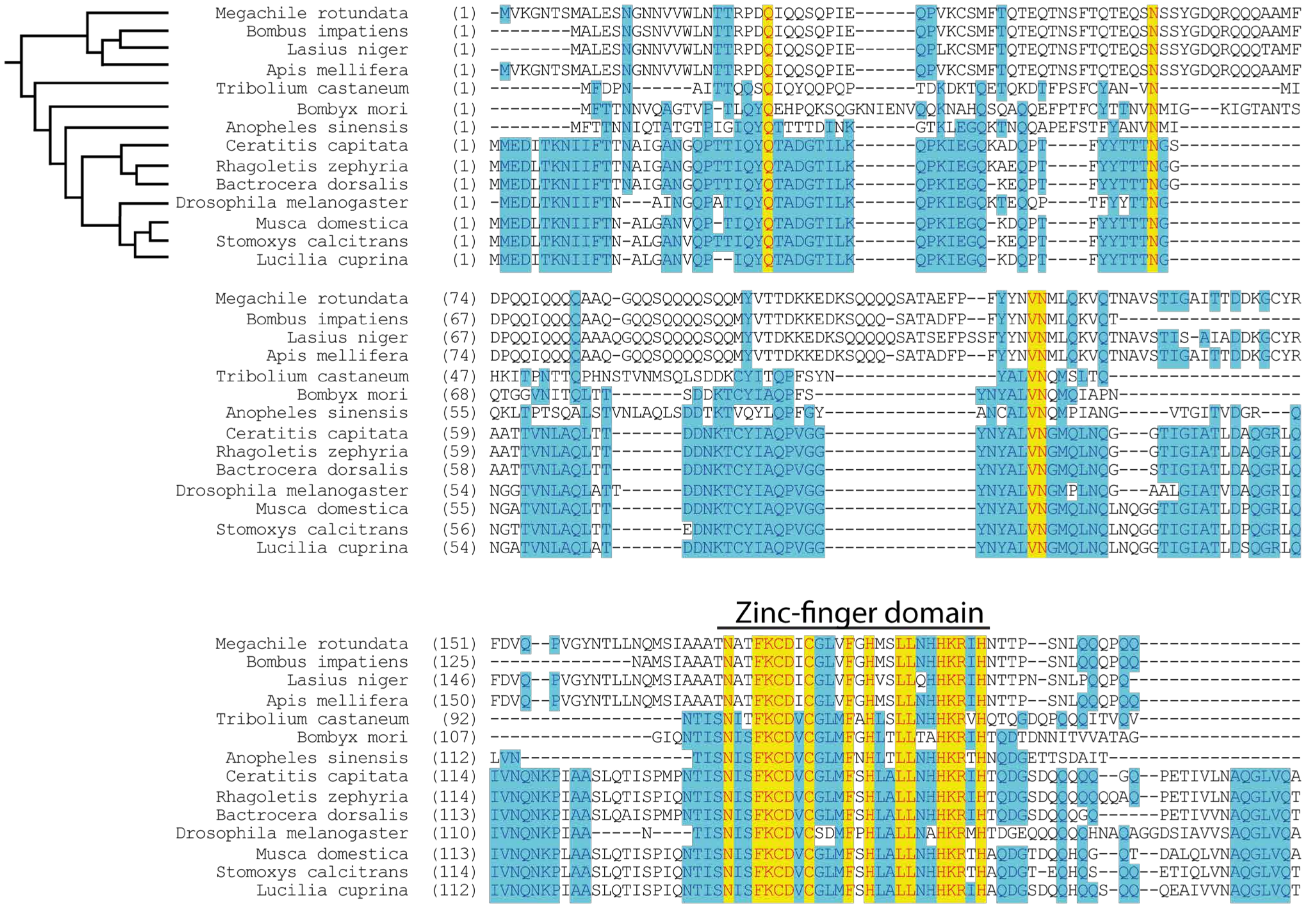

Supplementary Figure S5. Plots of the relaxation time for the ^{15}N nuclei of the backbone amide groups of $CLAMP^{40\text{-}153}$ and $CLAMP^{87\text{-}153}$ as a function of residue number. Shown are longitudinal relaxation time T_1, s (upper plot) and transverse relaxation time T_2, s (lower plot) measured at 700 MHz and 25 ºC. Error bars represent standard deviations estimated from duplicate relaxation time points.

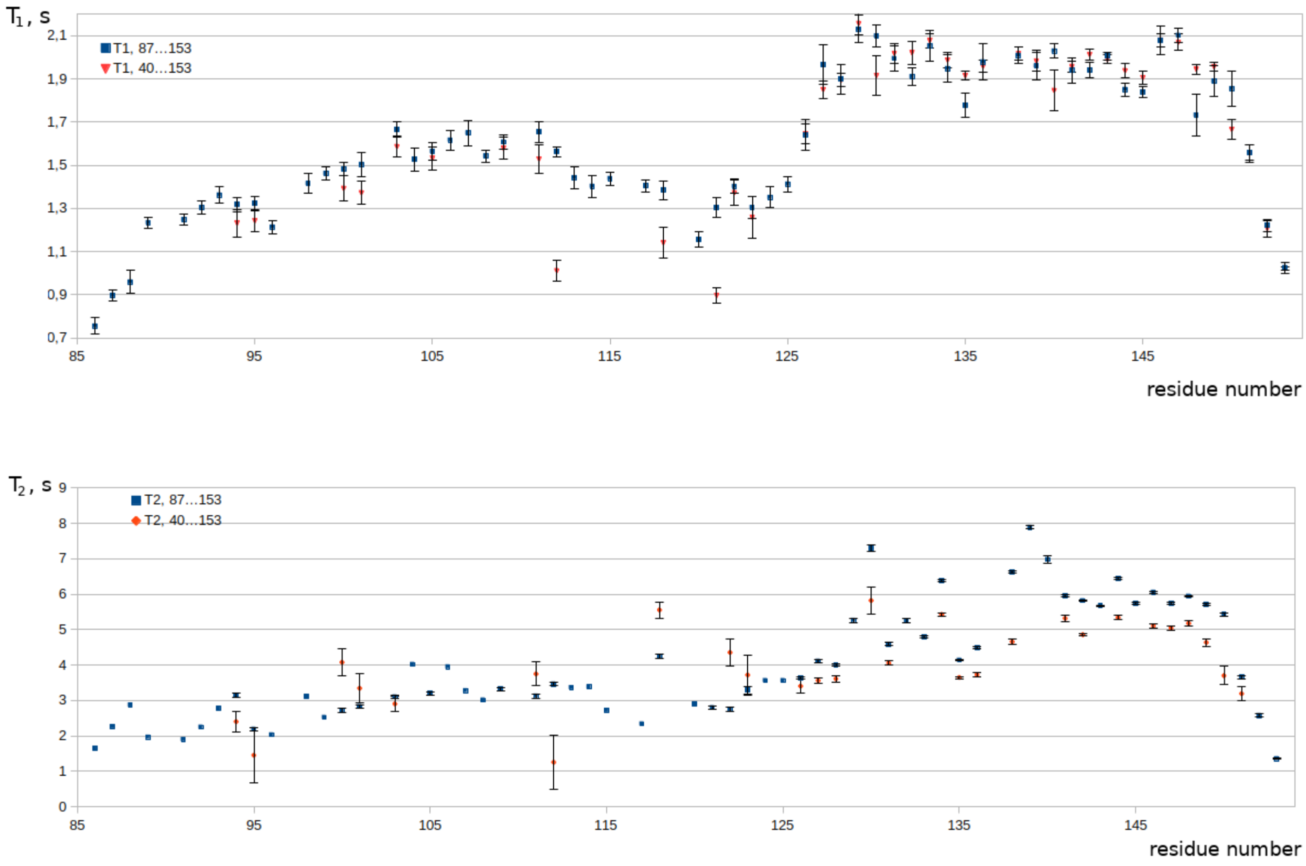

Supplementary Figure S6. ^{15}N,^{1}H SOFAST HMQC spectra of ^{15}N-labelled CLAMP$^{40\text{-}153}$ titrated with increasing concentrations of unlabeled MSL2$^{618\text{-}655}$ peptide. The spectra were recorded using NMR spectrometer Bruker Avance 600 MHz at 298K.

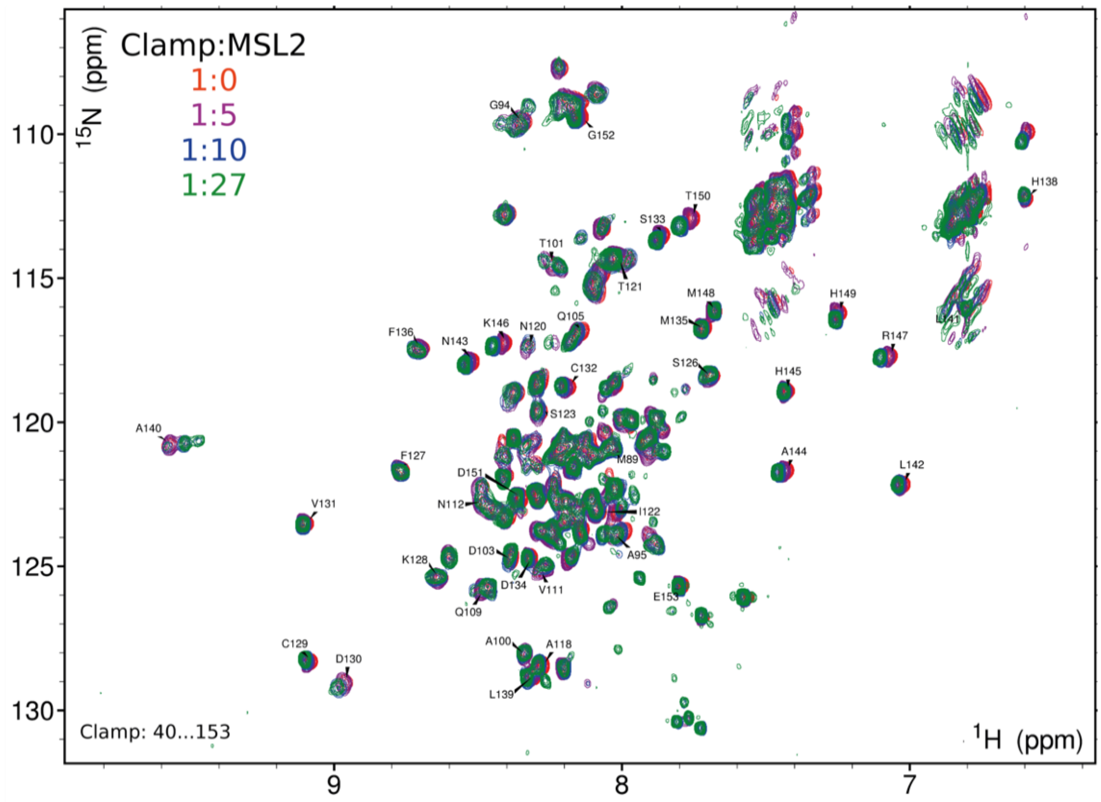

Supplementary Figure S7. Analysis of global C2H2 fingers amino-acid composition. **(A)** A logo representing sequence alignment and profile hidden Markov model for 109192 C2H2 zinc-fingers calculated with Skylign (20). **(B)** Probabilities of residues at known DNA-interacting positions are shown below.

A

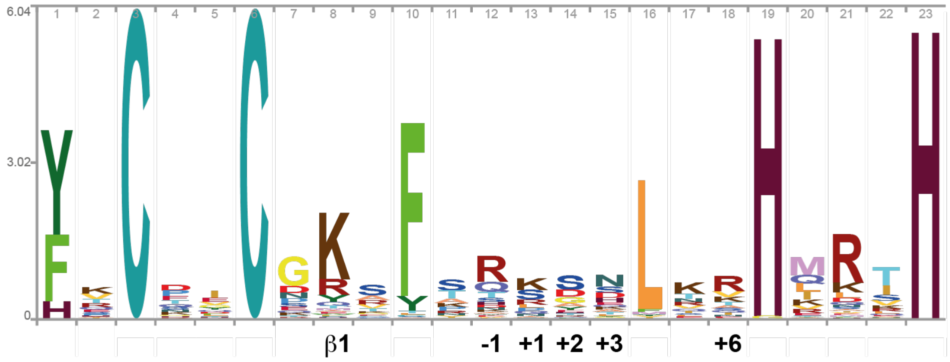

B

1

Residue	Probability	Residue	Probability	Residue	Probability	Residue	Probability
R	0.393	D	0.043	E	0.020	I	0.006
Q	0.156	N	0.040	V	0.018	W	0.005
T	0.091	K	0.033	L	0.017	M	0.005
S	0.057	A	0.026	F	0.009	C	0.004
H	0.048	Y	0.021	G	0.007	P	0.001

+1

Residue	Probability	Residue	Probability	Residue	Probability	Residue	Probability
K	0.259	Q	0.062	H	0.011	Y	0.006
S	0.224	N	0.050	V	0.008	F	0.006
R	0.110	A	0.039	M	0.008	D	0.004
L	0.072	T	0.034	G	0.007	W	0.004
P	0.072	E	0.012	I	0.007	C	0.004

+2

Residue	Probability	Residue	Probability	Residue	Probability	Residue	Probability
S	0.312	E	0.043	Q	0.016	P	0.008
D	0.153	H	0.036	F	0.015	I	0.007
G	0.138	T	0.034	K	0.013	L	0.006
A	0.075	N	0.020	C	0.013	W	0.006
Y	0.073	R	0.020	V	0.010	M	0.003

+3

Residue	Probability	Residue	Probability	Residue	Probability	Residue	Probability
N	0.248	Q	0.054	Y	0.028	I	0.005
S	0.137	K	0.054	G	0.023	F	0.005
D	0.109	A	0.051	M	0.014	C	0.004
H	0.103	T	0.049	R	0.014	W	0.002
E	0.055	L	0.033	V	0.008	P	0.002

+6

Residue	Probability	Residue	Probability	Residue	Probability	Residue	Probability
R	0.332	S	0.051	L	0.023	H	0.007
V	0.132	Q	0.051	D	0.013	C	0.002
K	0.109	I	0.045	Y	0.010	W	0.002
T	0.074	N	0.037	M	0.009	F	0.002
A	0.061	E	0.029	G	0.009	P	0.001

β1

Residue	Probability	Residue	Probability	Residue	Probability	Residue	Probability
K	0.613	M	0.017	F	0.008	N	0.005
R	0.143	A	0.015	S	0.007	P	0.004
Y	0.069	E	0.015	H	0.007	C	0.003
Q	0.036	W	0.012	V	0.006	G	0.002
T	0.022	L	0.009	I	0.006	D	0.002

Supplementary Figure S8. De novo prediction of possible DNA-binding by CLAMP N-terminal zinc-finger. **(A)** Position-weight matrix. **(B)** Schematic representation of DNA-binding site.

A

base	1	2	3
a	0.251	0.214	0.116
c	0.155	0.121	0.376
g	0.255	0.236	0.242
t	0.340	0.428	0.266

B

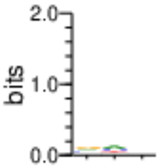

Supplementary Figure S9. Testing of the impact of point mutations on CLAMP-MSL2 interaction using yeast two-hybrid assay. Growth assay plates without histidine are shown (yeasts are unable to grow on this medium in the absence of interaction). AD stands for Activation Domain, BD – for DNA-Binding Domain of GAL4 protein.

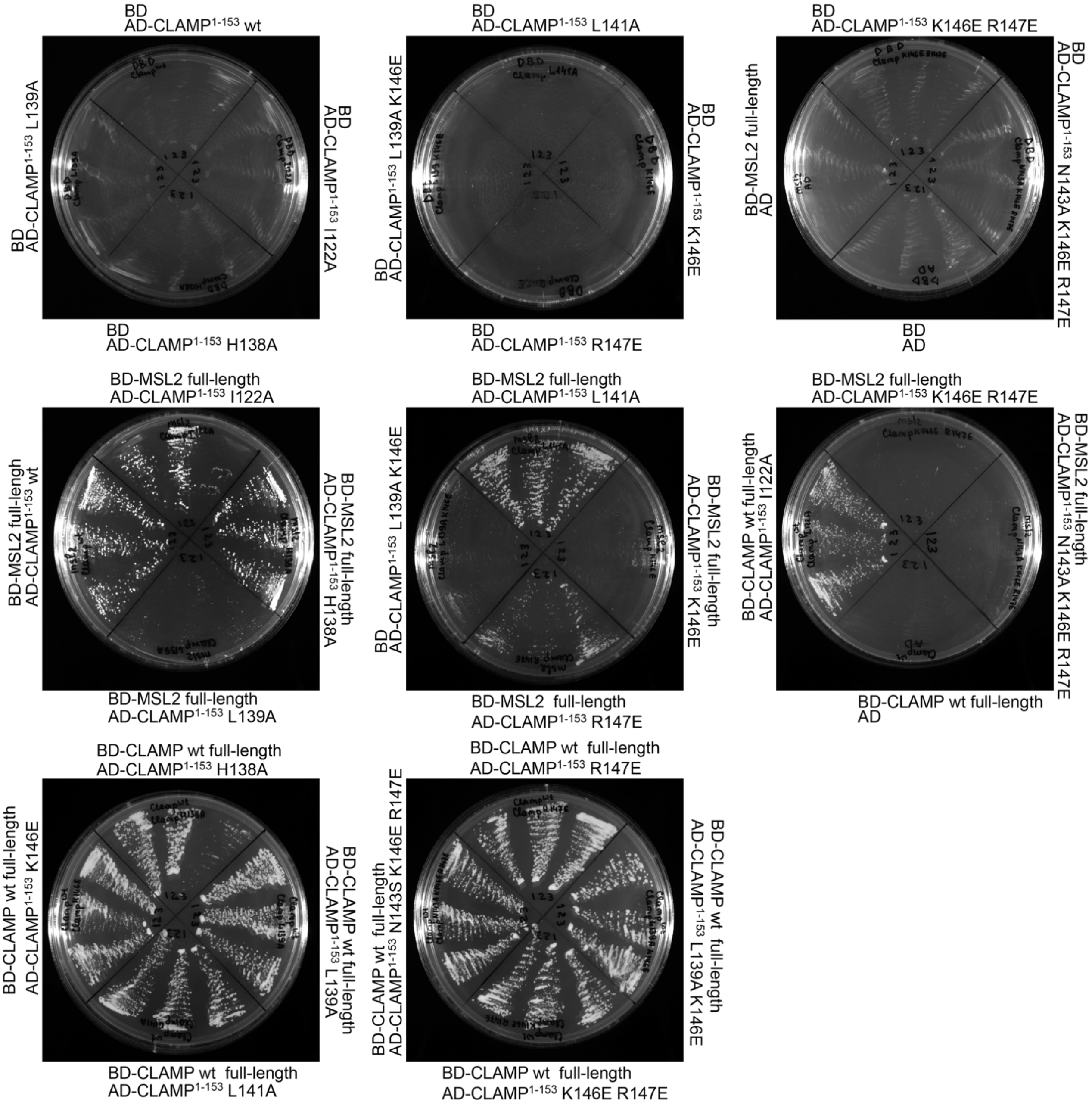

Supplementary Figure S10. 1D and 2D NMR spectra performed to validate the correct folding of CLAMP mutants.

A. The 1D NMR spectra of CLAMP$^{1\text{-}153}$ mutants (L141A, R147E, L139A, L139A/K146E, and N143R) in comparison with WT recorded using NMR spectrometer Bruker Avance 600 MHz at 298K.

B. ^{15}N,^{1}H HSQC spectra of 50 µM ^{15}N-labelled CLAMP$^{40\text{-}153}$ construct WT (magenta) and double mutant L139A/K146E (purple). Arrows indicate amid group signals of L139 and K146 residues on the CLAMP WT spectrum. The spectra were recorded using NMR spectrometer Bruker Avance 700 MHz at 298K.

A

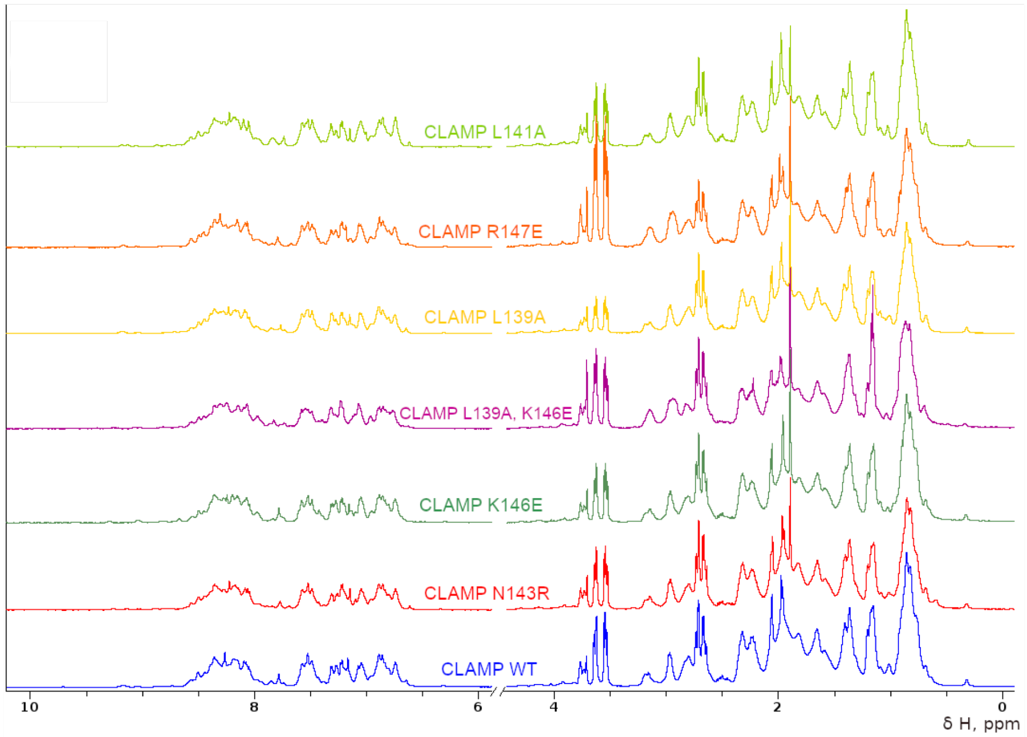

B

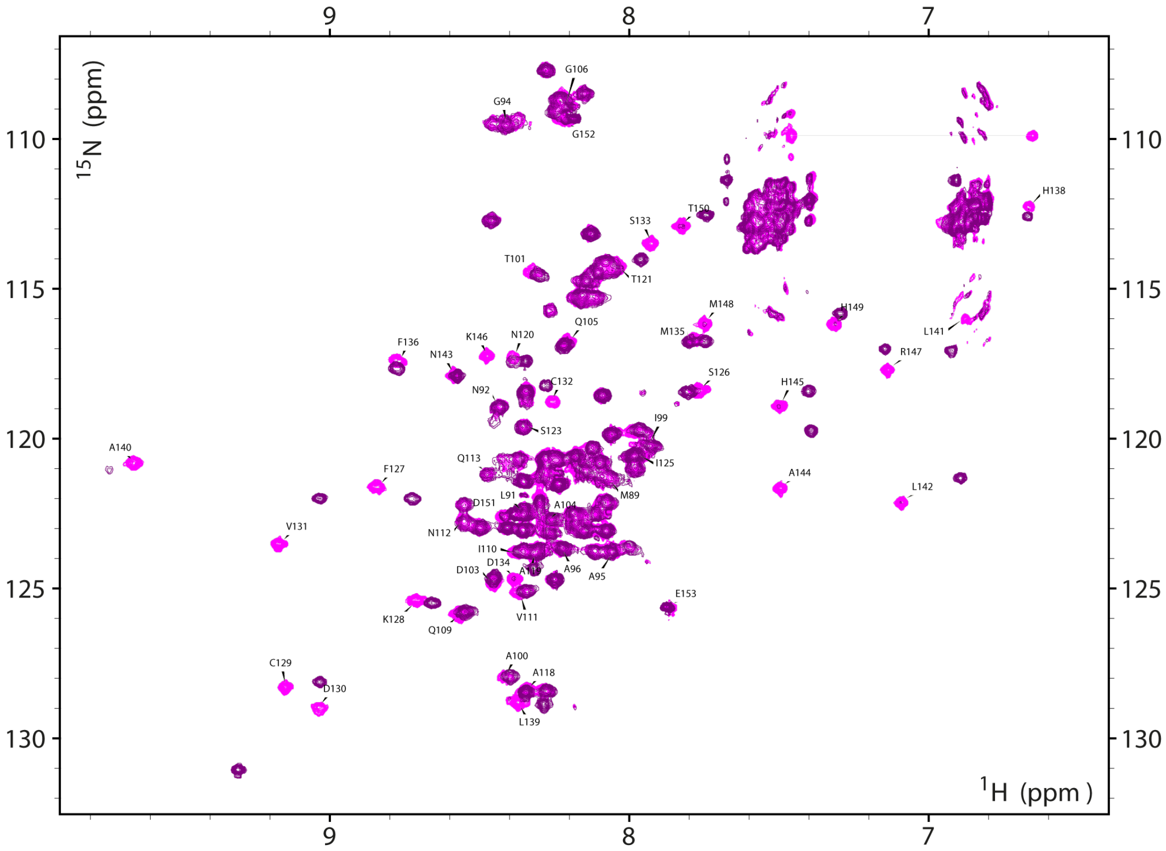

Supplementary Figure S11. ^{15}N,^{1}H HSQC spectra of 20 µM ^{15}N-labelled MSL2$^{618\text{-}655}$ (red), mixture of 20 µM ^{15}N-labelled MSL2$^{618\text{-}655}$ with 100 µM CLAMP$^{40\text{-}153}$ WT (green) and mixture of 20 µM ^{15}N-labelled MSL2$^{618\text{-}655}$ with 100 µM CLAMP$^{40\text{-}153}$ L139A K146E (black). The spectra were recorded using NMR spectrometer Bruker Avance 700 MHz at 298K.

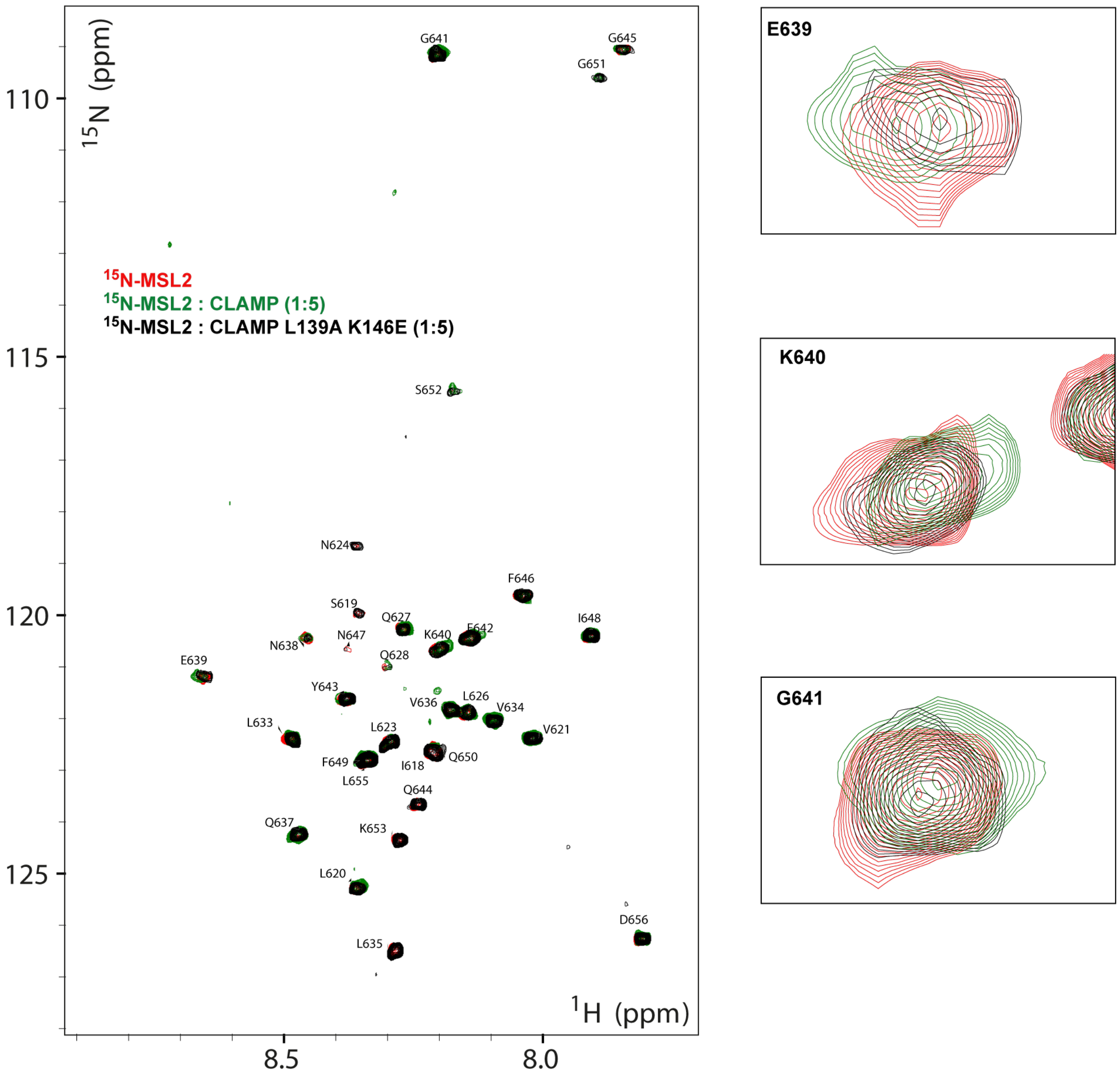

Supplementary Figure S12. Protein chain mobility of MSL2$^{618\text{-}655}$ represented by RCI-derived order parameter S^2 obtained from chemical shifts and the ANN-predicted secondary structure. Data obtained using Talos+ program (12).

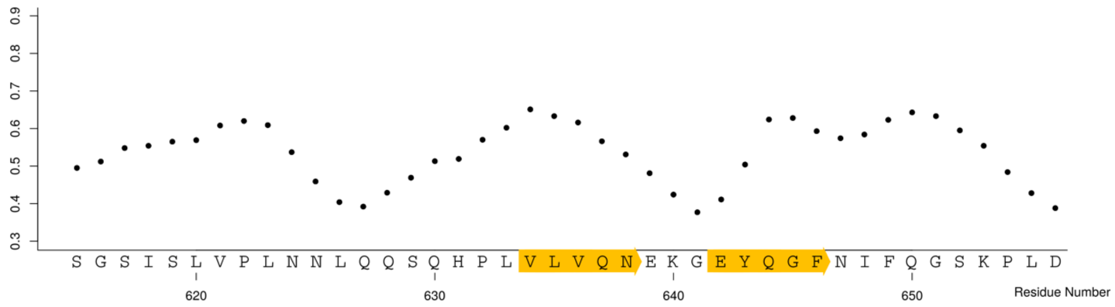

Supplementary Figure S13. ^{15}N-^{1}H SOFAST HMQC spectra of ^{15}N-labelled MSL2$^{618\text{-}655}$ titrated with increasing concentrations of unlabeled CLAMP$^{40\text{-}153}$ peptide. The spectra were recorded using NMR spectrometer Bruker Avance 600 MHz at 298K.

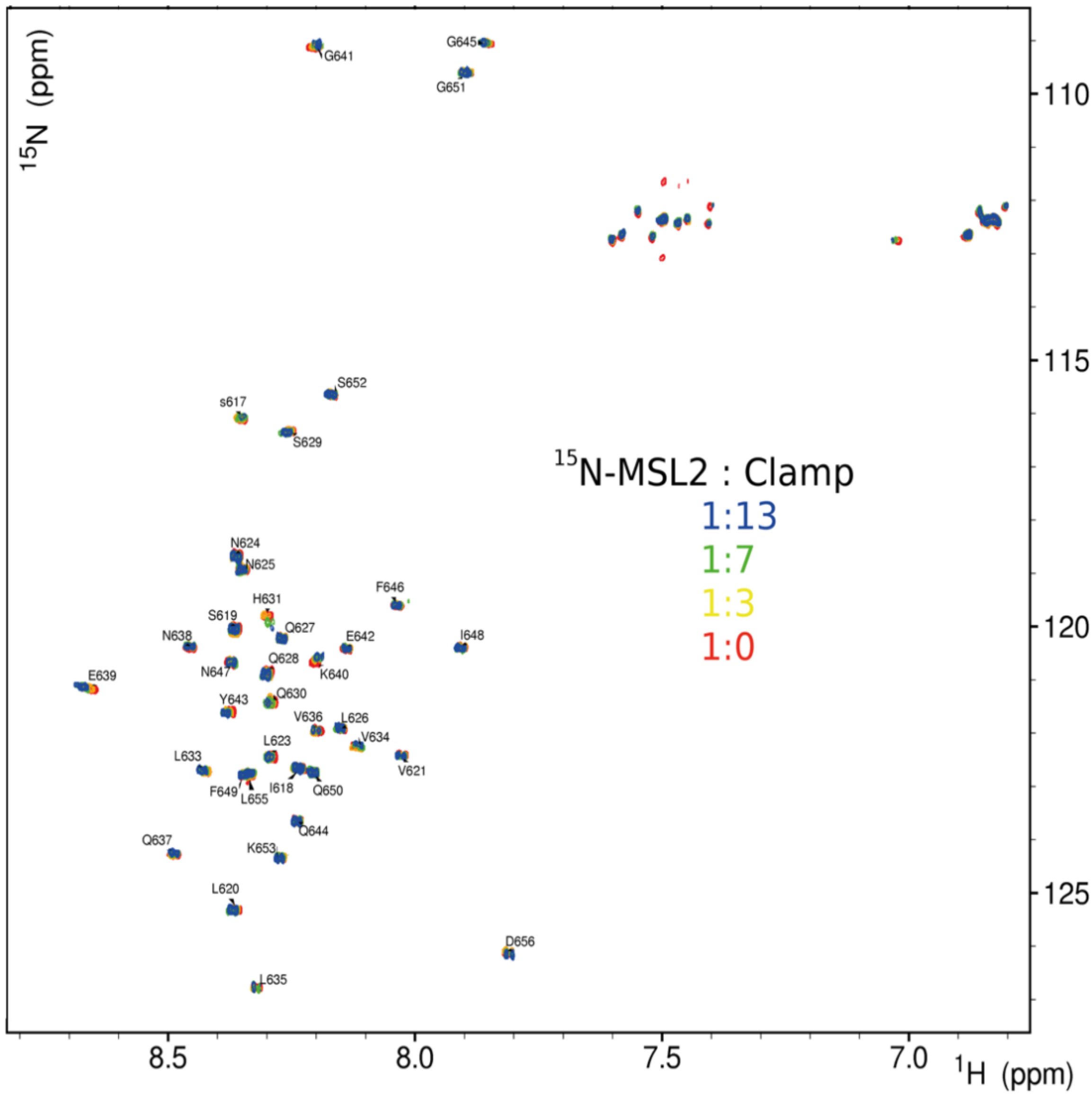

Supplementary Figure S14. The titration curves showing perturbation of chemical shifts of CLAMP$^{41\text{-}153}$ residues upon MSL2$^{618\text{-}655}$ binding. Black circles represent observed values of the chemical shifts, red lines represent the fitted curves calculated with the K_d values obtained by the nonlinear regression. The abscissa shows the concentration ratio MSL2:CLAMP, the ordinate shows the chemical shifts of amide protons of CLAMP. Concentrations of both interacting components were changed in the titration experiments and are shown at the bottom.

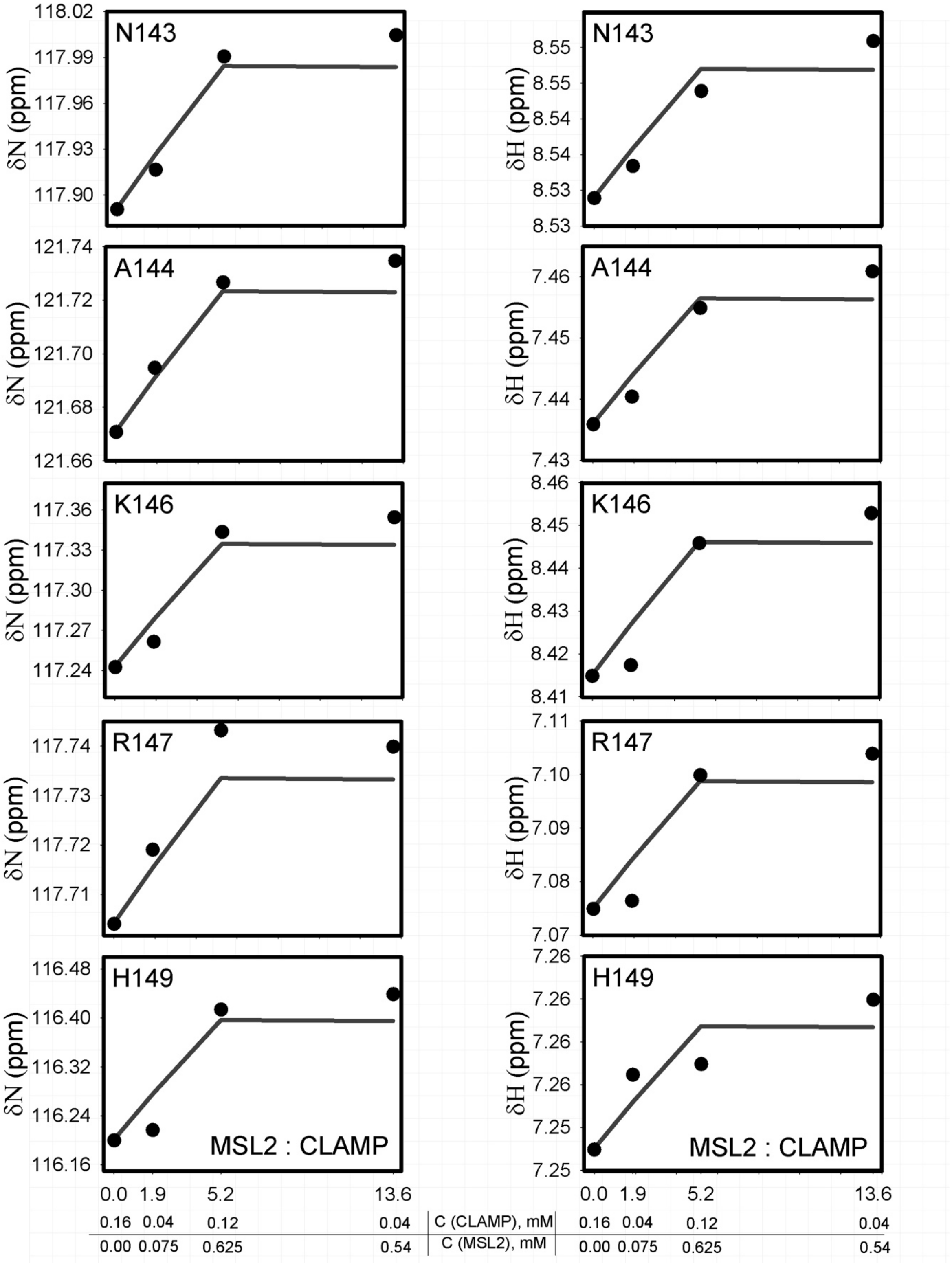

Supplementary Figure S15. Multiple sequence alignment of MSL2 CXC and C-terminal domains from various insects. CLAMP-interacting residues are shown in bold at the *D. melanogaster* MSL2 sequence. Identical residues are shown in yellow, conserved in green, blocks of identical – in blue.

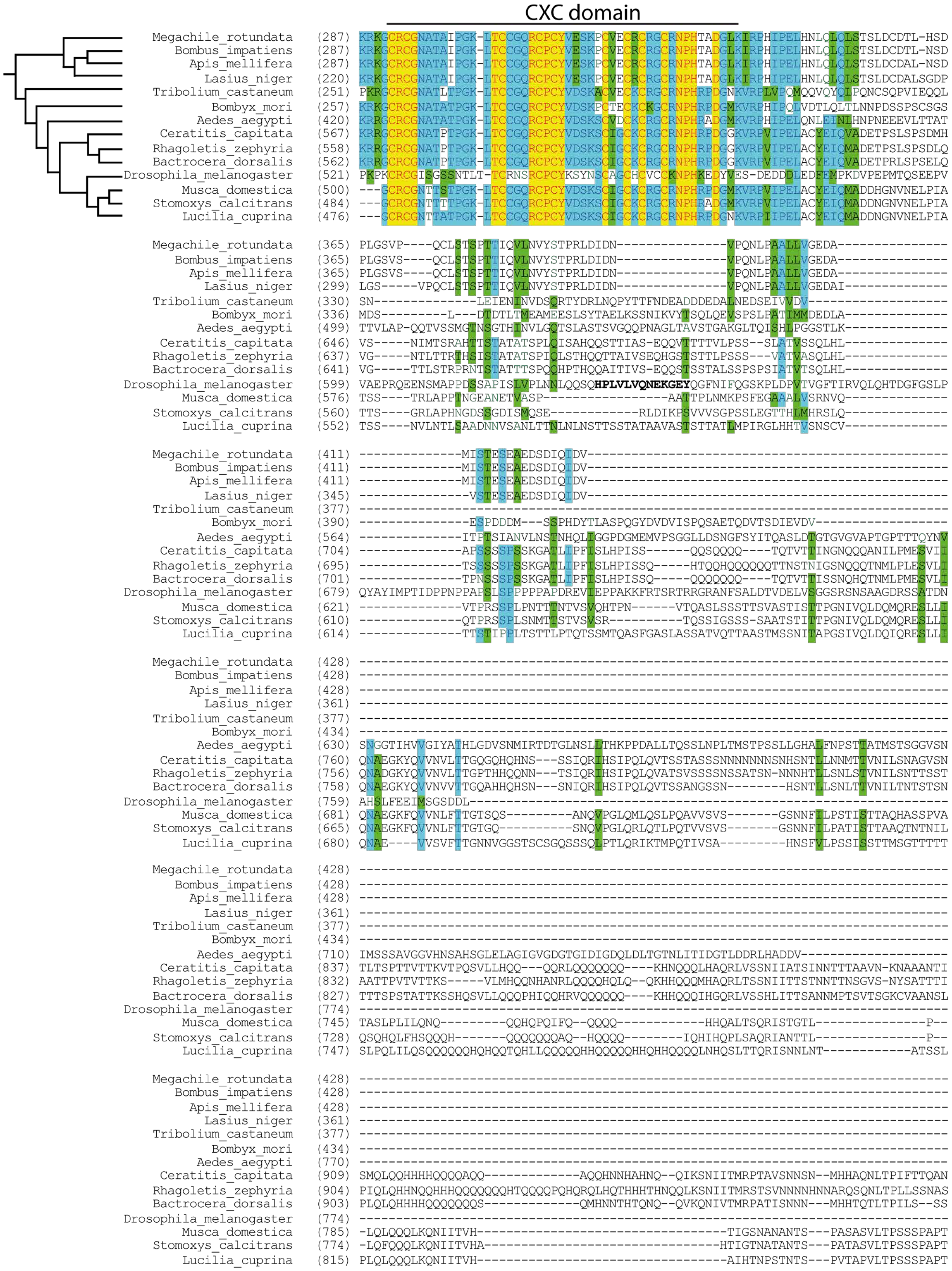

Supplementary Figure S16. (A) Interaction between 6xHis-tagged CLAMP and GST-tagged MSL2 proteins from honey bee (amCLAMP and amMSL2) and *D. melanogaster* studied with GST/6xHis-pulldown assay. **(B)** Interaction between 6xHis-tagged CLAMP and GST-tagged MSL2 proteins from *D. virilis* (dvMSL2) and *D. melanogaster* studied with GST/6xHis-pulldown assay.

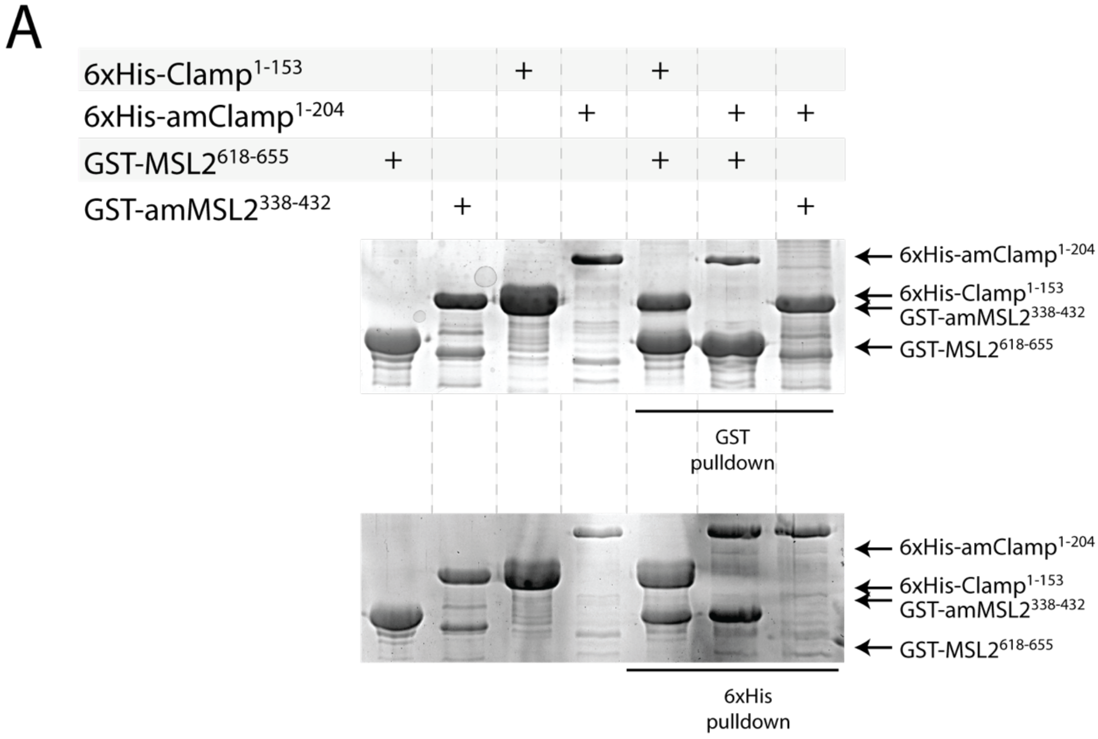

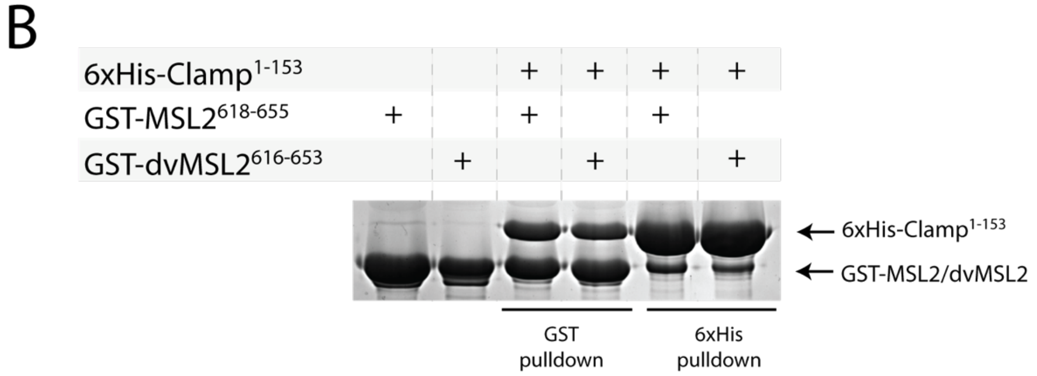

Supplementary Figure S17. **(A)** Western blots of protein extracts from transgenic flies expressing wild-type and mutant CLAMP proteins (left) and wild-type and mutant MSL2 proteins (right). **(B)** Relative roX2 expression levels in transgenic constructs normalized to rpl32 level.

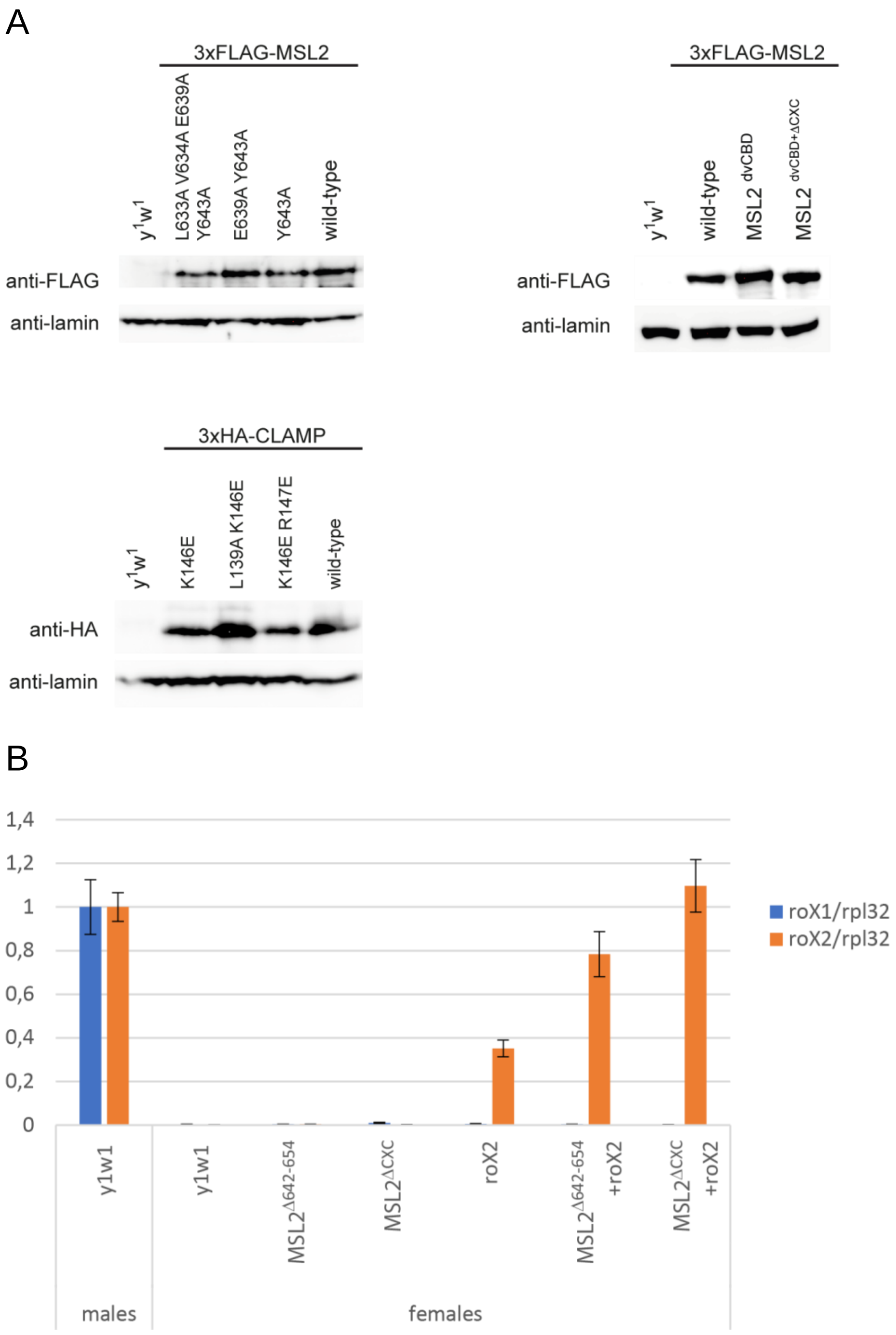

Supplementary Figure S18. **(A)** Effect of single amino-acid substitutions in the FLAG-tagged MSL2 proteins on DCC recruitment shown by immunostaining of polytene chromosomes with anti-FLAG and anti-MSL1 antibodies in females. **(B)** Effect of single amino-acid substitutions in the FLAG-tagged MSL2 protein on DCC recruitment shown by immunostaining of polytene chromosomes with MSL2 antibodies in females**.** Scale bar is 20 μm.

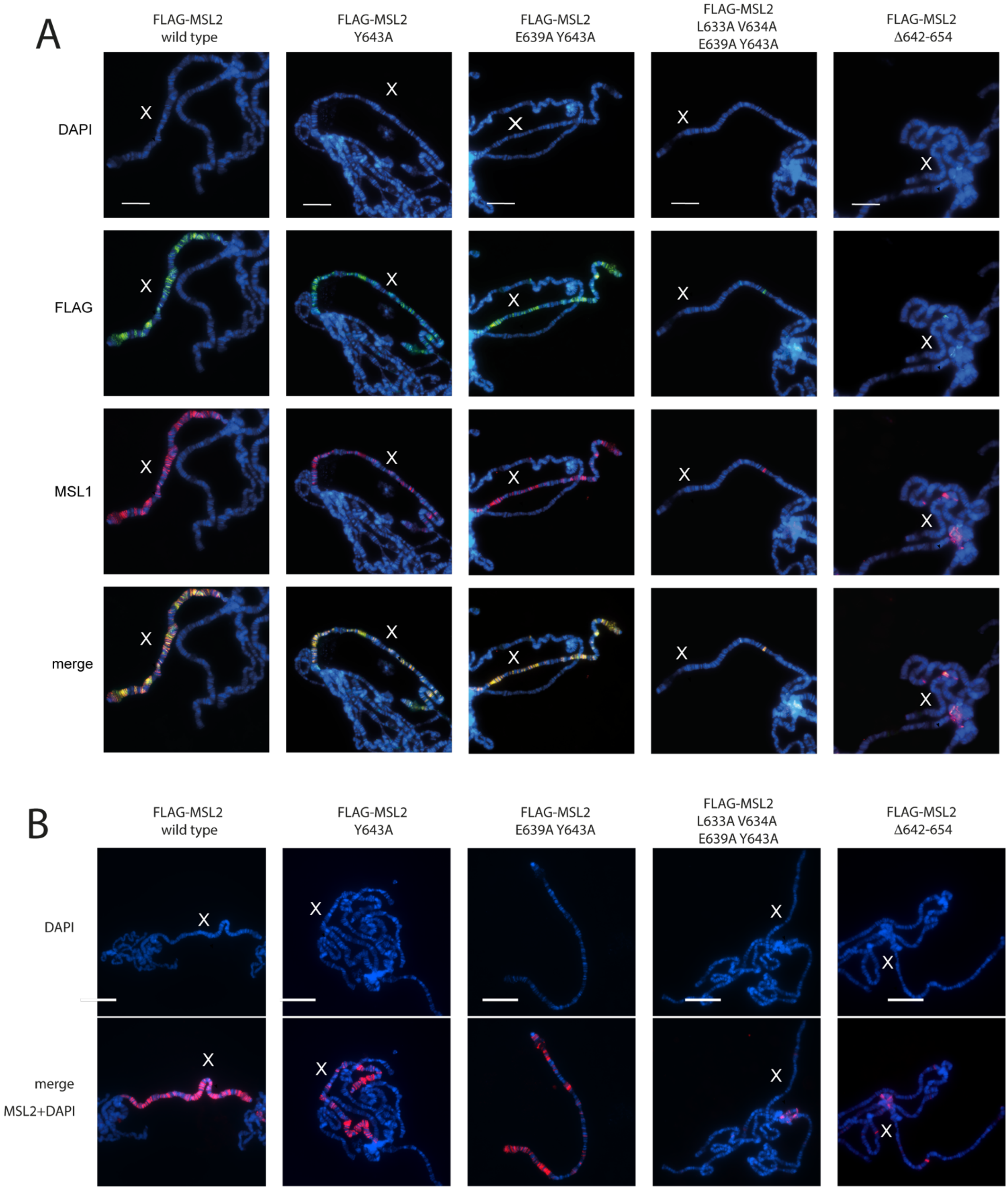

Supplementary Figure S19. Effect of replacement of the CLAMP binding domain with the homologous domain from D. virilis (dvCBD) and the deletion of the CXC domain in the FLAG-tagged MSL2 proteins on DCC recruitment shown by immunostaining of polytene chromosomes with anti-FLAG and anti-MSL1 antibodies in females. Scale bar is 20 μm.

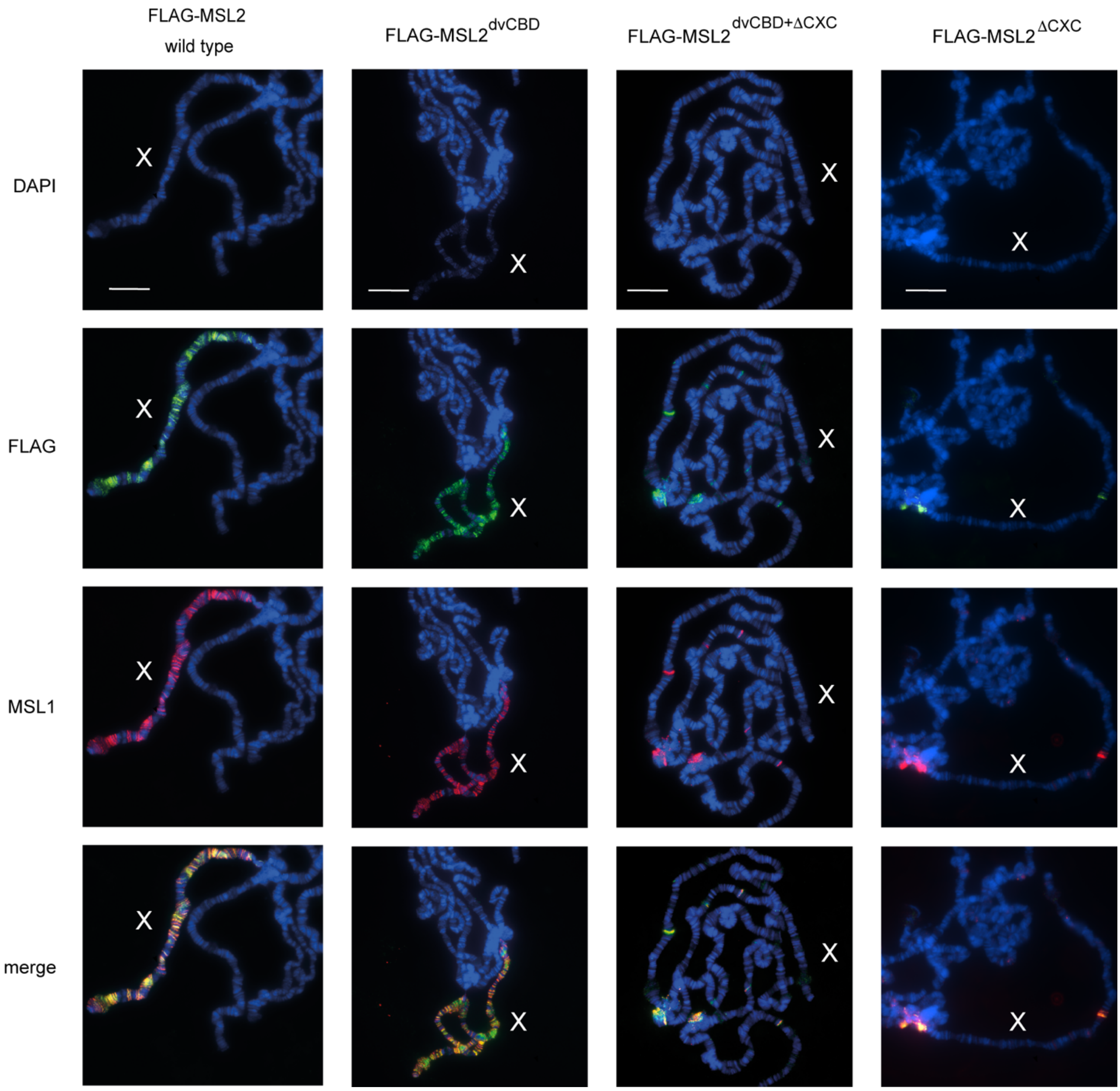

Supplementary Figure S20. Effect of RoX RNA overexpression on DCC recruitment in females expressing FLAG-tagged MSL2 proteins bearing mutations within CXC and CLAMP-binding domains shown by immunostaining of polytene chromosomes with anti-FLAG and anti-MSL1 antibodies in females. Scale bar is 20 μm.

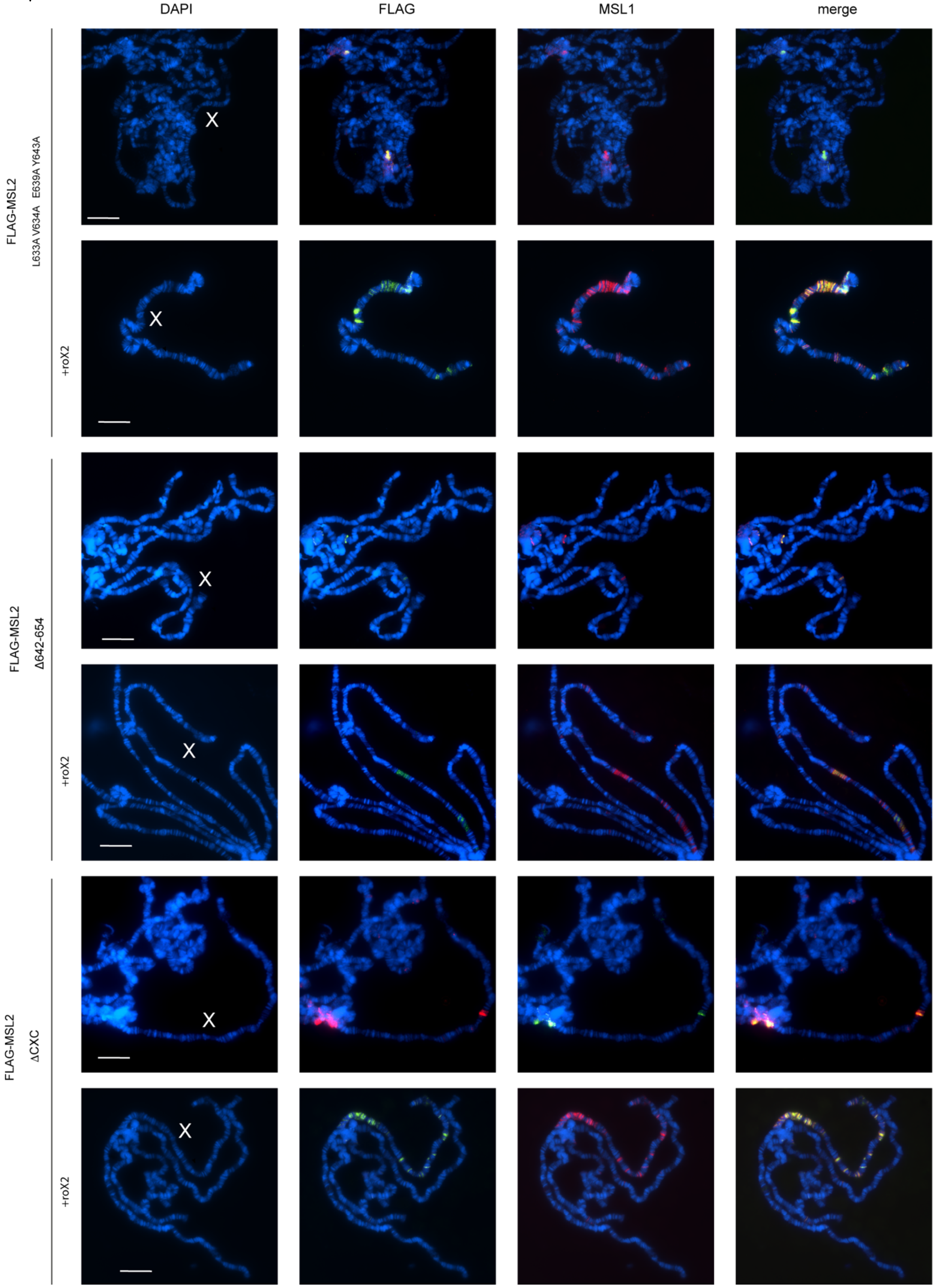

Supplementary Figure S21. Schematic representations of interactions mediated by C2H2 zinc-fingers: intramolecular (upper row), and interactions mediated by UBZ-type fingers (lower row). Cartoons were drawn according to following structures (PDB ID): 2GLI (Gli F1 – F2 (21)), 1NCS (Swi5 (22)), 6DF5 (Kaiso F1 and F3 (23)), 3WWQ (FAAP20 UBZ – Ubiquitin (24)), 3VHT (WRNIP UBZ – Ubiquitin (25)).

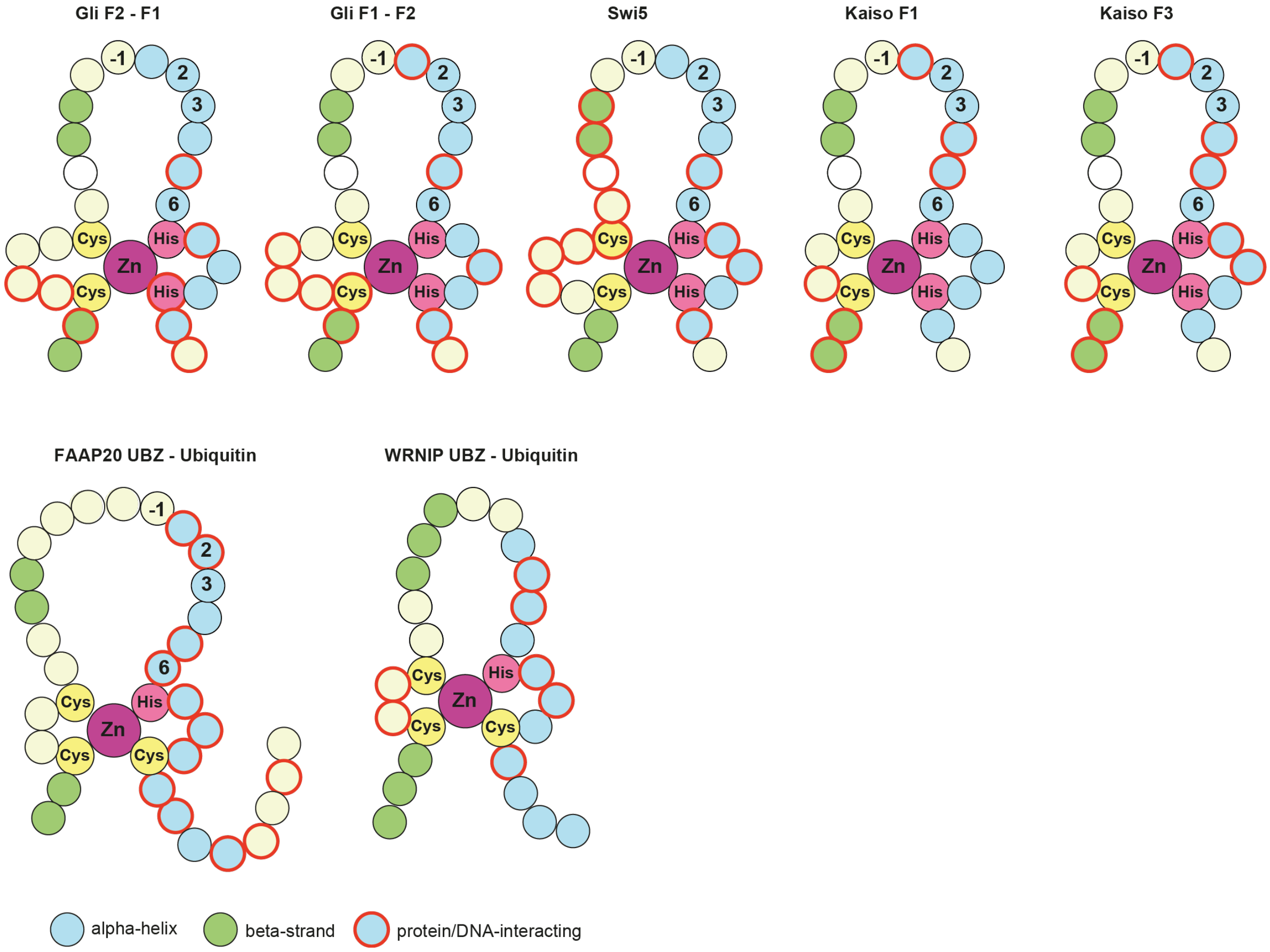

SUPPLEMENTARY REFERENCES

1. Bischof, J., Maeda, R.K., Hediger, M., Karch, F. and Basler, K. (2007) An optimized transgenesis system for Drosophila using germ-line-specific phiC31 integrases. *Proc Natl Acad Sci U S A*, **104**, 3312-3317.
2. Sabirov, M., Kyrchanova, O., Pokholkova, G.V., Bonchuk, A., Klimenko, N., Belova, E., Zhimulev, I.F., Maksimenko, O. and Georgiev, P. (2021) Mechanism and functional role of the interaction between CP190 and the architectural protein Pita in Drosophila melanogaster. *Epigenetics Chromatin*, **14**, 16.
3. Tikhonova, E., Fedotova, A., Bonchuk, A., Mogila, V., Larschan, E.N., Georgiev, P. and Maksimenko, O. (2019) The simultaneous interaction of MSL2 with CLAMP and DNA provides redundancy in the initiation of dosage compensation in Drosophila males. *Development*, **146**.
4. El-Gebali, S., Mistry, J., Bateman, A., Eddy, S.R., Luciani, A., Potter, S.C., Qureshi, M., Richardson, L.J., Salazar, G.A., Smart, A. *et al.* (2019) The Pfam protein families database in 2019. *Nucleic Acids Res*, **47**, D427-D432.
5. Vandevenne, M., Jacques, D.A., Artuz, C., Nguyen, C.D., Kwan, A.H., Segal, D.J., Matthews, J.M., Crossley, M., Guss, J.M. and Mackay, J.P. (2013) New insights into DNA recognition by zinc fingers revealed by structural analysis of the oncoprotein ZNF217. *J Biol Chem*, **288**, 10616-10627.
6. Gupta, A., Christensen, R.G., Bell, H.A., Goodwin, M., Patel, R.Y., Pandey, M., Enuameh, M.S., Rayla, A.L., Zhu, C., Thibodeau-Beganny, S. *et al.* (2014) An improved predictive recognition model for Cys(2)-His(2) zinc finger proteins. *Nucleic Acids Res*, **42**, 4800-4812.
7. Persikov, A.V., Wetzel, J.L., Rowland, E.F., Oakes, B.L., Xu, D.J., Singh, M. and Noyes, M.B. (2015) A systematic survey of the Cys2His2 zinc finger DNA-binding landscape. *Nucleic Acids Res*, **43**, 1965-1984.
8. Pavletich, N.P. and Pabo, C.O. (1991) Zinc finger-DNA recognition: crystal structure of a Zif268-DNA complex at 2.1 A. *Science*, **252**, 809-817.
9. Persikov, A.V. and Singh, M. (2014) De novo prediction of DNA-binding specificities for Cys2His2 zinc finger proteins. *Nucleic Acids Res*, **42**, 97-108.
10. Delaglio, F., Grzesiek, S., Vuister, G.W., Zhu, G., Pfeifer, J. and Bax, A. (1995) NMRPipe: a multidimensional spectral processing system based on UNIX pipes. *J Biomol NMR*, **6**, 277-293.
11. Lee, W., Tonelli, M. and Markley, J.L. (2015) NMRFAM-SPARKY: enhanced software for biomolecular NMR spectroscopy. *Bioinformatics*, **31**, 1325-1327.
12. Shen, Y., Delaglio, F., Cornilescu, G. and Bax, A. (2009) TALOS plus : a hybrid method for predicting protein backbone torsion angles from NMR chemical shifts. *Journal of Biomolecular Nmr*, **44**, 213-223.
13. EngineeringToolBox. (2004), https://www.engineeringtoolbox.com/water-dynamic-kinematic-viscosity-d_596.html,
14. Linge, J.P., Habeck, M., Rieping, W. and Nilges, M. (2003) ARIA: automated NOE assignment and NMR structure calculation. *Bioinformatics*, **19**, 315-316.
15. Brunger, A.T., Adams, P.D., Clore, G.M., DeLano, W.L., Gros, P., Grosse-Kunstleve, R.W., Jiang, J.S., Kuszewski, J., Nilges, M., Pannu, N.S. *et al.* (1998) Crystallography & NMR system: A new software suite for macromolecular structure determination. *Acta Crystallogr D*, **54**, 905-921.
16. Shen, Y., Vernon, R., Baker, D. and Bax, A. (2009) De novo protein structure generation from incomplete chemical shift assignments. *Journal of Biomolecular Nmr*, **43**, 63-78.
17. Schanda, P., Kupce, E. and Brutscher, B. (2005) SOFAST-HMQC experiments for recording two-dimensional heteronuclear correlation spectra of proteins within a few seconds. *J Biomol NMR*, **33**, 199-211.
18. Polshakov, V.I., Batuev, E. A., Mantsyzov, A. B. (2019) NMR screening and studies of target–ligand interactions. *Russ Chem Rev.* , **88**, 59-98.
19. Misof, B., Liu, S., Meusemann, K., Peters, R.S., Donath, A., Mayer, C., Frandsen, P.B., Ware, J., Flouri, T., Beutel, R.G. *et al.* (2014) Phylogenomics resolves the timing and pattern of insect evolution. *Science*, **346**, 763-767.

20. Wheeler, T.J., Clements, J. and Finn, R.D. (2014) Skylign: a tool for creating informative, interactive logos representing sequence alignments and profile hidden Markov models. *BMC Bioinformatics*, **15**, 7.
21. Pavletich, N.P. and Pabo, C.O. (1993) Crystal structure of a five-finger GLI-DNA complex: new perspectives on zinc fingers. *Science*, **261**, 1701-1707.
22. Dutnall, R.N., Neuhaus, D. and Rhodes, D. (1996) The solution structure of the first zinc finger domain of SWI5: a novel structural extension to a common fold. *Structure*, **4**, 599-611.
23. Nikolova, E.N., Stanfield, R.L., Dyson, H.J. and Wright, P.E. (2020) A Conformational Switch in the Zinc Finger Protein Kaiso Mediates Differential Readout of Specific and Methylated DNA Sequences. *Biochemistry*, **59**, 1909-1926.
24. Toma, A., Takahashi, T.S., Sato, Y., Yamagata, A., Goto-Ito, S., Nakada, S., Fukuto, A., Horikoshi, Y., Tashiro, S. and Fukai, S. (2015) Structural basis for ubiquitin recognition by ubiquitin-binding zinc finger of FAAP20. *PLoS One*, **10**, e0120887.
25. Suzuki, N., Rohaim, A., Kato, R., Dikic, I., Wakatsuki, S. and Kawasaki, M. (2016) A novel mode of ubiquitin recognition by the ubiquitin-binding zinc finger domain of WRNIP1. *FEBS J*, **283**, 2004-2017.
